# Effects of exercise interventions on fall-related functional performance in postmenopausal women with osteoporosis or low bone mass: a systematic review and meta-analysis with an exploratory analysis of reported consistency with ACSM recommendations

**DOI:** 10.3389/fphys.2026.1903705

**Published:** 2026-08-12

**Authors:** Shixian Li, Neng Pan, Lulu Niu, Min Yao, Shuai Zhao, Chenming Liu, Dongying Li

**Affiliations:** 1 School of Physical Education, Yunnan Minzu University, Kunming, Yunnan, China; 2 Faculty of Physical Culture, Gdansk University of Physical Education and Sport, Gdansk, Poland; 3 School of Physical Education, Yunnan University, Kunming, Yunnan, China; 4 Kunming Medical University, Kunming, Yunnan, China

**Keywords:** ACSM, exercise intervention, fall-related functional performance, low bone mass, osteoporosis, postmenopausal women

## Abstract

**Objective:**

To evaluate the effects of exercise on fall-related functional performance in postmenopausal women with osteoporosis or low bone mass and to explore whether intervention effects differed according to the reported consistency of the exercise prescription with American College of Sports Medicine (ACSM) recommendations.

**Methods:**

Six electronic databases were searched from inception to 3 May 2026. Immediate post-intervention mean differences for the Timed Up and Go (TUG) test, Berg Balance Scale (BBS), and 30-second sit-to-stand test (30STS) were pooled using inverse-variance random-effects models with restricted maximum likelihood estimation, Hartung–Knapp inference, and prediction intervals. Prespecified exercise-prescription components were classified as meeting, unclear or unreported, or not meeting the ACSM criteria.

**Results:**

Eighteen trials contributed 21 intervention arms. Exercise improved TUG performance (MD = −1.57 s, 95% CI −2.52 to −0.63; I² = 88.5%) and 30STS performance (MD = 3.88 repetitions, 95% CI 1.93 to 5.83; I² = 87.6%). The estimated effect on BBS was highly uncertain (MD = 3.37 points, 95% CI −2.68 to 9.43; I² = 83.6%). Formal tests found no evidence of a difference between the high and low/uncertain reported-consistency groups for TUG (p = 0.733), BBS (p = 0.985), or 30STS (p = 0.262). Prediction intervals crossed the null for all three outcomes. Certainty of evidence was low for TUG and 30STS and very low for BBS.

**Conclusion:**

Exercise may improve functional mobility and repeated sit-to-stand performance, whereas the effect on BBS remains highly uncertain. The available analyses do not demonstrate superior effects for interventions with higher reported consistency with ACSM recommendations; the subgroup findings are exploratory.

**Systematic Review Registration:**

https://www.crd.york.ac.uk/PROSPERO/view/CRD420261353752, identifier CRD420261353752.

## 1 Introduction

Accelerated bone loss after menopause increases women’s susceptibility to osteoporosis and fragility fractures. Oestrogen deficiency promotes bone resorption, and continuing changes in bone mass and microarchitecture reduce skeletal strength. Skeletal disease may also be accompanied by reduced lower-limb strength, impaired postural control, gait instability, fear of falling, and limitations in activities of daily living, which together increase the likelihood and consequences of falls (Cummings and Melton, 2002; Kanis et al., 2013; Cosman et al., 2014; Eastell et al., 2019; Camacho et al., 2020; North American Menopause Society, 2021).

Fall-related capacity is multidimensional and cannot be fully represented by a single clinical test. The TUG records the sequence of standing up from a chair, walking, turning, and sitting down again and is used to assess functional mobility. The BBS assesses performance in static and dynamic balance tasks. The 30STS reflects lower-limb functional strength and endurance through the number of repeated stands completed in 30 s (Berg et al., 1989, 1992; Podsiadlo and Richardson, 1991; Jones et al., 1999; Rikli and Jones, 1999; Bohannon, 2006). Together, these tests capture complementary aspects of independent living and fall-related physical function in women with osteoporosis or low bone mass.

Exercise is an important component of the non-pharmacological management of osteoporosis. Relevant guidelines commonly combine progressive resistance, balance, and weight-bearing or impact activities and emphasise safety and progression to improve skeletal and physical function (Giangregorio et al., 2014, 2015; Beck et al., 2017; Exercise Is Medicine, 2021). However, responses to an intervention may also be influenced by programme type, weekly frequency, intensity, duration, adherence, and supervision (Martyn-St James and Carroll, 2008; Howe et al., 2011; Zhao et al., 2015; Shojaa et al., 2020; Kistler-Fischbacher et al., 2021; Hoffmann et al., 2023).

Interventions in this field are markedly heterogeneous in their training emphasis, dose, progression, and reporting detail; grouping them simply as ‘exercise’ may therefore obscure clinically relevant differences. Comparing reported prescription components with ACSM recommendations offers a structured but exploratory means of describing this variation. This approach evaluates the exercise prescription reported in each publication rather than participants’ actual adherence or intervention fidelity.

Previous reviews have synthesised the effects of exercise on bone health, falls, and physical function in postmenopausal or osteoporotic populations, but have generally classified interventions by exercise type rather than systematically examining how reported prescription components correspond to a single osteoporosis exercise framework (Howe et al., 2011; Giangregorio et al., 2014, 2015; Hoffmann et al., 2023). We selected the ACSM/Exercise is Medicine recommendations because they provide concise and clinically interpretable criteria for weight-bearing/aerobic, resistance, and flexibility exercise. Our researcher-developed scoring method was intended to explore heterogeneity in reporting and prescription dose, not to establish that ACSM recommendations are superior to other guidelines.

Accordingly, this review pooled randomised evidence on immediate post-intervention TUG, BBS, and 30STS performance in postmenopausal women with osteoporosis or low bone mass and explored differences between interventions with high and low/uncertain reported consistency with ACSM recommendations.

## 2 Materials and methods

This review was reported in accordance with PRISMA 2020, and the protocol was prospectively registered in PROSPERO (CRD420261353752) (Page et al., 2021). The registration specified an ACSM-based assessment of exercise prescription but did not fully prespecify the component weights, 65% classification threshold, distinction between unclear reporting and clear non-fulfilment, or the sensitivity analyses. These operational details were specified before the revised analyses and are reported transparently here as protocol clarifications.

### 2.1 Search strategy

Six electronic databases were searched from inception to 3 May 2026: PubMed, Embase, Web of Science, Scopus, the Cochrane Library, and CINAHL via EBSCO. Clinical trial registries were not searched separately. Reference lists of included studies, relevant systematic reviews, and guidelines were not searched for additional eligible studies. The search combined controlled vocabulary and free-text terms for osteoporosis or low bone mass, postmenopausal women, exercise, and randomised or controlled clinical trials. The final executable strategy and number of records retrieved for each database are provided in **Supplementary Table 1**.

### 2.2 Eligibility criteria

Studies were eligible if they: (1) used a randomised controlled design; (2) enrolled postmenopausal women with osteoporosis, osteopenia, or low bone mass as defined by bone mineral density or clinical diagnostic criteria in the original study; (3) evaluated an exercise intervention; (4) used usual activity, health education, standard care, or no structured exercise as the comparator; (5) reported immediate post-intervention sample size, mean, and standard deviation for TUG, BBS, or 30STS; and (6) were published as full-text, peer-reviewed journal articles, irrespective of publication language. Studies of mixed populations were eligible only when data for the target postmenopausal osteoporosis/osteopenia population could be extracted separately or when the entire sample met the target-population criteria.

We excluded non-randomised studies, reviews, conference abstracts, case reports, and protocols; mixed populations that were ineligible or could not be separated; exercise combined with nutrition or another co-intervention; exercise compared with another structured exercise programme; studies without usable outcome or variance data; and reports that duplicated participants included elsewhere. Grey literature was excluded; however, full-text, peer-reviewed journal articles were eligible regardless of publication language.

### 2.3 Study selection and data extraction

Two reviewers SL and DL independently screened titles and abstracts and assessed potentially eligible full texts. Disagreements were resolved through discussion, with NP acting as a third reviewer when necessary. Rayyan was used solely to identify duplicate records; every suggested duplicate was checked manually, and no automated eligibility-exclusion rule or automation flag was used.

SL and DL independently extracted study characteristics and outcome data, resolving discrepancies through discussion and, when required, adjudication by NP. Extracted information included author, year, country, sample size, participant characteristics, age, intervention, comparator, programme duration, prescription details, and outcomes. Only immediate post-intervention assessments were extracted; follow-up measurements were not combined with immediate post-intervention data. Because adjusted estimates and variances of change scores were reported inconsistently across trials, post-intervention means and standard deviations were used. Baseline comparability was examined descriptively from the original reports. Missing standard deviations were not imputed, and outcomes without usable variance estimates were excluded from the synthesis. Where data could not be extracted, every effort was made to obtain the original data from the corresponding authors.

For multi-arm trials in which several eligible exercise arms shared the same control group, the control-group sample size was divided equally across the relevant comparisons, while its mean and standard deviation were left unchanged. This avoided double counting and unit-of-analysis errors while retaining all eligible intervention arms.

### 2.4 Assessment of reported consistency with ACSM recommendations

The assessment was based on the ACSM/Exercise is Medicine resource Being Active When You Have Osteoporosis and a scoring framework developed for this review. Component weights and the 65% threshold were determined by the researchers and were neither directly specified nor validated by ACSM. The framework evaluates the exercise prescription reported in the literature; it does not assess participant adherence, intervention fidelity, supervision quality, programme implementation, compliance, or safety. The ACSM/Exercise is Medicine reference criteria used for this assessment are presented in **Table 1**.

**Table 1:** ACSM/Exercise is Medicine reference criteria for osteoporosis exercise prescription.

Exercise domain	Prescription component	Reference criterion
Balance-oriented weight-bearing/aerobic activity	Frequency	4–5 d/wk for weight-bearing activity; ≥3 d/wk for aerobic activity.
	Intensity	Moderate-to-high intensity, or balance postures held for 15–30 s.
	Duration	30–60 impacts/d; 20–45 min/d of weight-bearing activity; or 30–60 min/session of aerobic activity.
Resistance training	Frequency	2–3 d/wk
	Repetitions	8–12 reps
	Sets	Progress to 2 sets after approximately 2 weeks.
Flexibility training	Frequency	5–7 d/wk
	Intensity	Stretch to tightness; hold for 10–30 s, or 30–60 s for older adults.

ACSM, American College of Sports Medicine; d/wk, days per week; min, minutes; reps, repetitions; s, seconds.

The weighting scheme was determined by the researchers before data extraction, with reference to the ACSM/Exercise is Medicine osteoporosis exercise recommendations and related clinical evidence. The ACSM criteria were used to judge whether each dose component met the recommendation, whereas the weights reflected the researchers’ judgement of the relative importance of components within the same exercise domain.

The assessment comprised three operational domains: balance-oriented weight-bearing/aerobic activity, resistance training, and flexibility training. Within the balance-oriented weight-bearing/aerobic domain, frequency, intensity, and duration were weighted at 30%, 35%, and 35%, respectively. Intensity and duration received relatively greater weight because bone adaptation is closely related to adequate mechanical loading and exercise intensity, while weight-bearing and balance training may also contribute to skeletal loading and fall-risk management (Exercise Is Medicine, 2021; Kistler-Fischbacher et al., 2021). Within the resistance-training domain, frequency, repetitions, and sets were weighted at 30%, 40%, and 30%, respectively. Because load intensity was not a separate component in the scoring framework, repetitions were used as an indirect indicator of training dose and effort; relevant guidelines also emphasise resistance and balance training for maintaining function and reducing fall risk (Giangregorio et al., 2014; Exercise Is Medicine, 2021). Within the flexibility domain, frequency and intensity/hold time were weighted at 40% and 60%, respectively, with the latter component incorporating both stretch intensity and hold time (Giangregorio et al., 2014; Exercise Is Medicine, 2021). These operational groupings and specific weights were researcher-defined for comparison of heterogeneous exercise prescriptions and are not ACSM-prescribed or validated scoring weights.

The domain score was calculated as follows:


$$C_{d}=\sum_{i=1}^{m_{d}}\left(w_{di}\times x_{di}\right)$$

where C_d is the weighted score for domain d, w_di is the prespecified weight for component i (summing to 100% within each domain), and x_di is the indicator used in the primary analysis. Each component was first coded as M (the reported prescription met the criterion), U (unclear or unreported), or N (the reported prescription clearly did not meet the criterion). In the primary analysis, x_di = 1 for M and x_di = 0 for U or N. Although U and N both received zero in the primary calculation, they were retained as distinct descriptive categories to avoid misrepresenting incomplete reporting as clear non-fulfilment.

The total score was calculated as follows:


$$C_{\text{total}}=\frac{1}{k}\sum_{d=1}^{k}C_{d}$$

where C_total is the mean score across the k exercise domains included in the intervention. Domains not included in an intervention were excluded from the denominator. If a domain was included but a prescription component was unclear or unreported, that component was coded U and received zero in the primary analysis. Two reviewers (SL and DL) scored the interventions independently, and unresolved disagreements were adjudicated by NP.

Because no validated threshold exists, C_total ≥65% was defined as high reported consistency and C_total<65% as low/uncertain reported consistency. Robustness analyses increased the threshold to 70%; assigned U half of the corresponding prespecified component weight while retaining full weight for M and zero for N; excluded studies at high risk of bias; and omitted one study arm at a time. We also considered excluding all intervention arms with insufficient prescription reporting, but too few comparisons remained for stable subgroup synthesis.

### 2.5 Risk of bias and certainty of evidence

SL and DL independently applied the Cochrane RoB 2 tool to outcome-specific results for TUG, BBS, and 30STS. Where an intervention arm had identical judgements across several outcomes, these were displayed jointly in the summary figure; disagreements were resolved with the involvement of a third reviewer NP. The domains assessed were the randomisation process, deviations from intended interventions, missing outcome data, measurement of the outcome, and selection of the reported result. GRADE was used to assess certainty of evidence for each outcome across risk of bias, inconsistency, indirectness, imprecision, and publication bias (Sterne et al., 2019; Higgins et al., 2024).

### 2.6 Statistical analysis

Analyses were conducted in R version 4.5.3, with readxl and dplyr used for data management. For each outcome, meta::metacont() pooled post-intervention mean differences using inverse-variance random-effects models. Between-study variance was estimated by restricted maximum likelihood. Confidence intervals used the Hartung–Knapp method with the *ad hoc* standard-error safeguard (adhoc.hakn.ci = “se”), and prediction intervals were calculated. Heterogeneity was summarised using τ², I², and Cochran’s Q. Random-effects tests for subgroup differences compared the high and low/uncertain reported-consistency classifications.

Sensitivity analyses used a 70% alternative threshold, half-weight scoring for U, exclusion of studies at outcome-specific high risk of bias, and leave-one-study-arm-out analyses. Exploratory univariable meta-regressions for each available moderator were fitted separately with metafor::rma.uni(); multivariable models were not fitted because few effect estimates were available. Funnel plots were inspected visually, and Egger’s regression and Begg–Mazumdar rank-correlation tests were calculated using metafor::regtest() and ranktest(). All subgroup, sensitivity, small-study-effect, and meta-regression analyses were considered exploratory.

## 3 Results

### 3.1 Study selection

**Figure 1:**
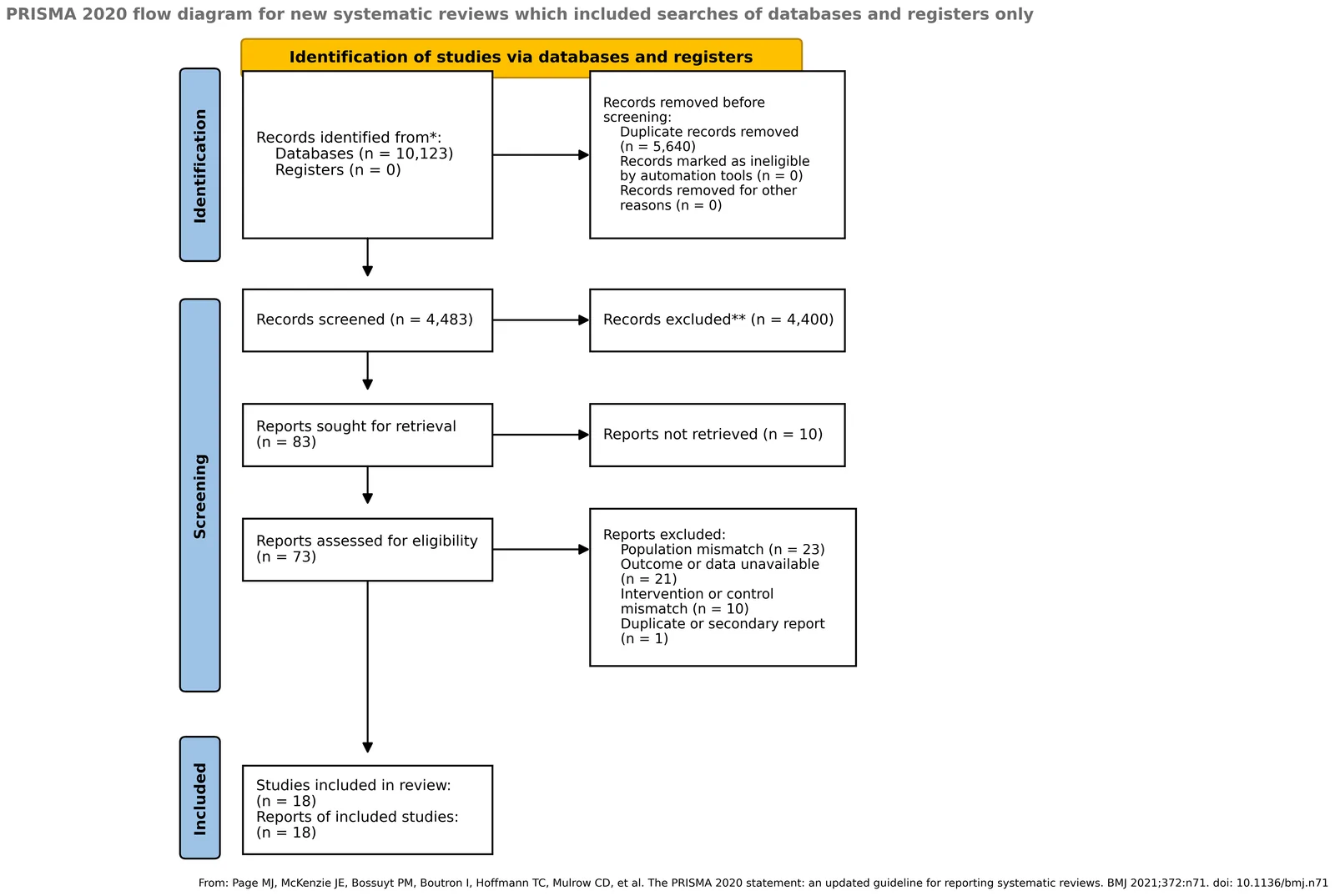
PRISMA 2020 flow diagram of study selection.

The six database searches yielded 10,123 records. Rayyan identified 5,640 potential duplicate records, all of which were checked manually before removal. After screening 4,483 titles and abstracts, 4,400 records were excluded. We sought 83 reports for full-text retrieval; 10 could not be retrieved, and 73 were assessed for eligibility. Fifty-five full-text reports were excluded because of an ineligible population (n = 23), missing outcome or usable data (n = 21), an ineligible intervention or comparator (n = 10), or a duplicate/secondary report (n = 1). Excluded full-text reports and reasons for exclusion are listed in **Supplementary Table 2**. Eighteen reports, representing 18 randomised trials, were included (**Figure 1**).

### 3.2 Study characteristics

The 18 randomised trials included 1,181 participants from 15 countries or regions: Türkiye (Alkan et al., 2011; Cergel et al., 2019), Russia (Evstigneeva et al., 2016), Japan (Kanemaru et al., 2010), Greece (Kotsili et al., 2025), Taiwan, China (Wen et al., 2017; Lee et al., 2021), Brazil (Madureira et al., 2007; Teixeira et al., 2010), India (Mewara and Chhajed, 2024), Hungary (Mikó et al., 2018), Egypt (Mohsen et al., 2016), Portugal (Monteiro et al., 2026), Poland (Ossowski et al., 2016), Spain (Otero et al., 2017), Austria (Posch et al., 2019), Pakistan (Riaz et al., 2024), and Norway (Stanghelle et al., 2020). Outcome-specific syntheses included 17 study arms for TUG (471 participants in exercise groups and 410 in control groups), eight arms for BBS (194 and 162 participants, respectively), and eight arms for 30STS (280 and 271 participants, respectively). Interventions included aerobic, balance, multicomponent, and resistance programmes. Study and intervention-arm characteristics are presented in **Supplementary Table 3**.

### 3.3 Reported consistency with ACSM recommendations

Each intervention arm was scored for the exercise-prescription components it included using the ACSM/Exercise is Medicine osteoporosis resource and the weighting rules developed for this review. Scores of ≥65% were classified as high reported consistency and scores of<65% as low/uncertain reported consistency. Of 21 intervention arms, 10 were classified as high (≥65%) and 11 as low/uncertain (<65%). Component-level M, U, and N judgements and weighted scores are reported separately in **Supplementary Table 4**.

### 3.4 Risk of bias

**Figure 2:**
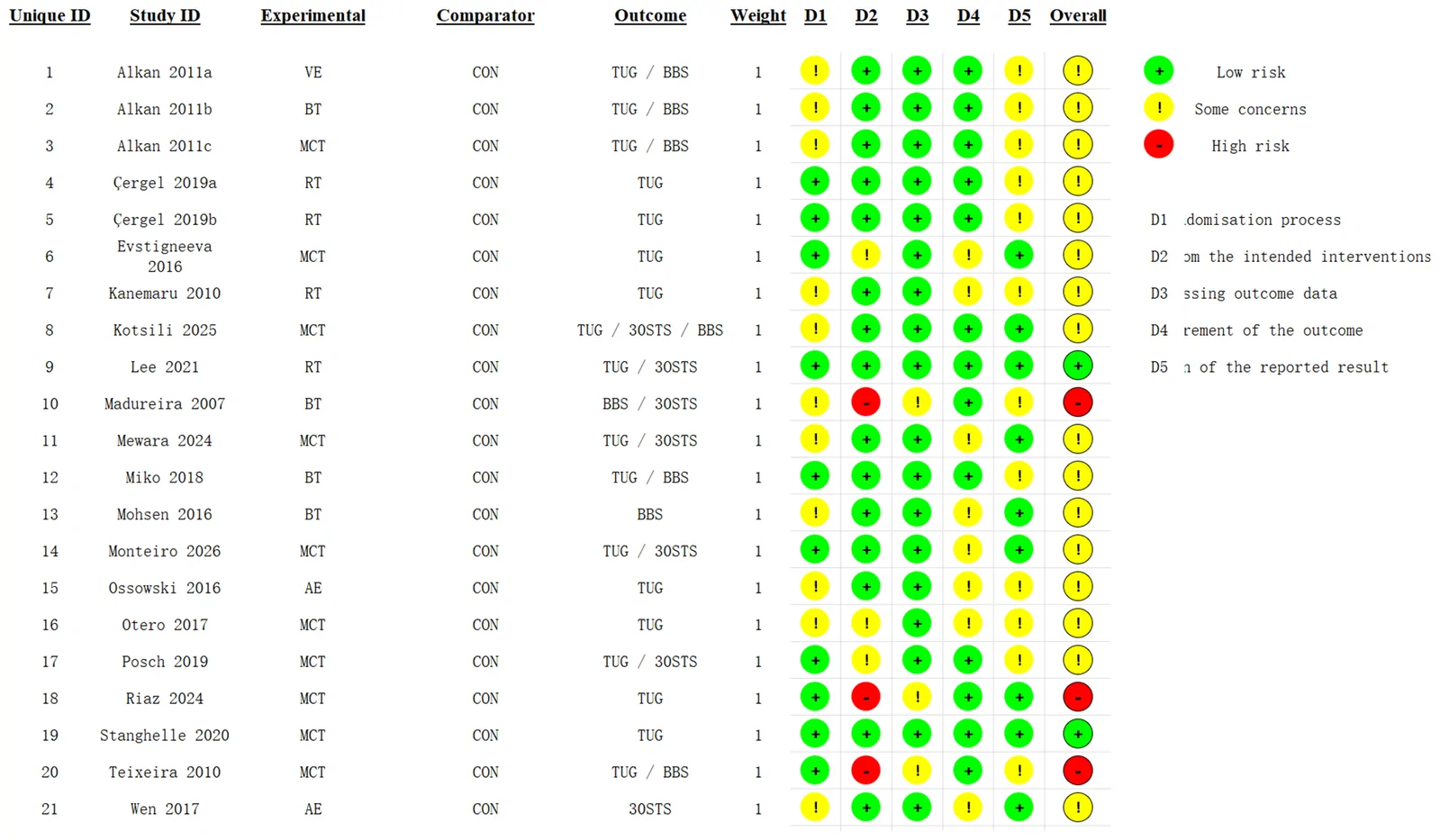
RoB 2 traffic-light plot.

**Figure 3:**
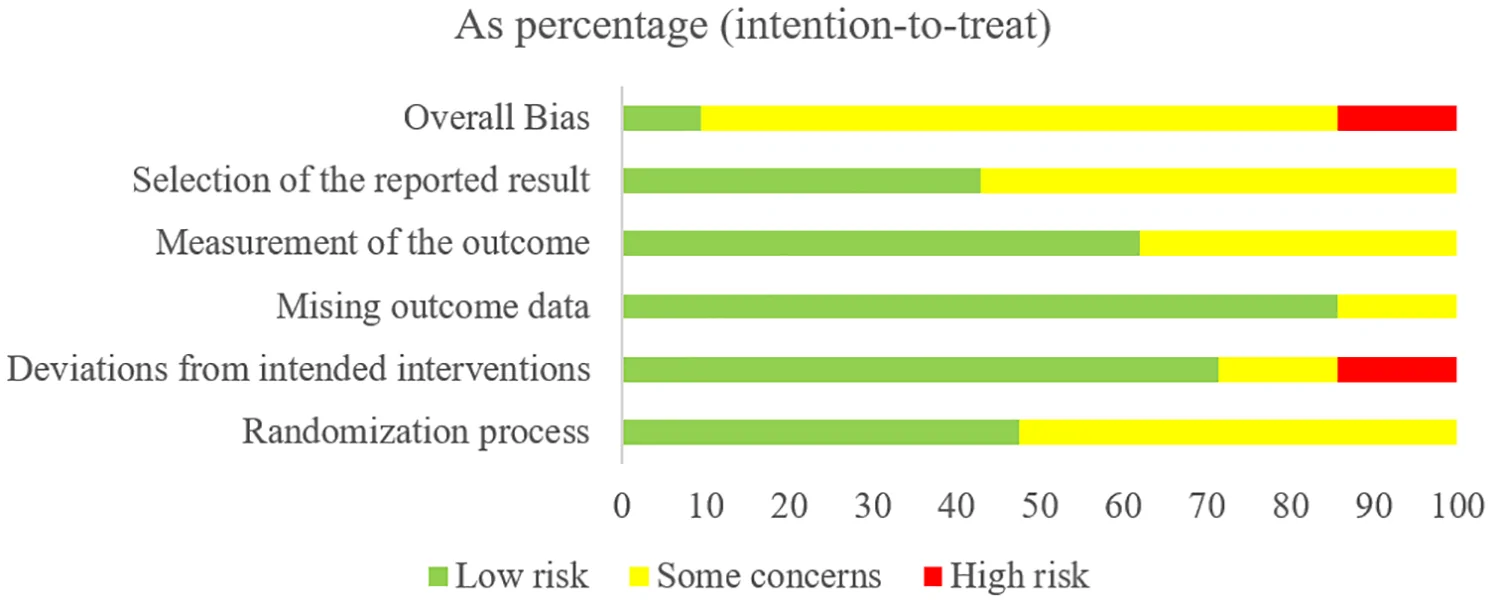
Summary of RoB 2 judgements.

Among the 21 displayed intervention-arm judgements from 18 studies, two were at low risk of bias, 16 raised some concerns, and three were at high risk. High risk arose primarily from deviations from intended interventions; concerns were also identified in the randomisation process, outcome measurement, missing outcome data, and selection of the reported result. Outcome-specific RoB 2 judgements are presented in **Figures 2** and **3**.

### 3.5 Timed Up and Go: overall and reported-consistency subgroup effects

**Figure 4:**
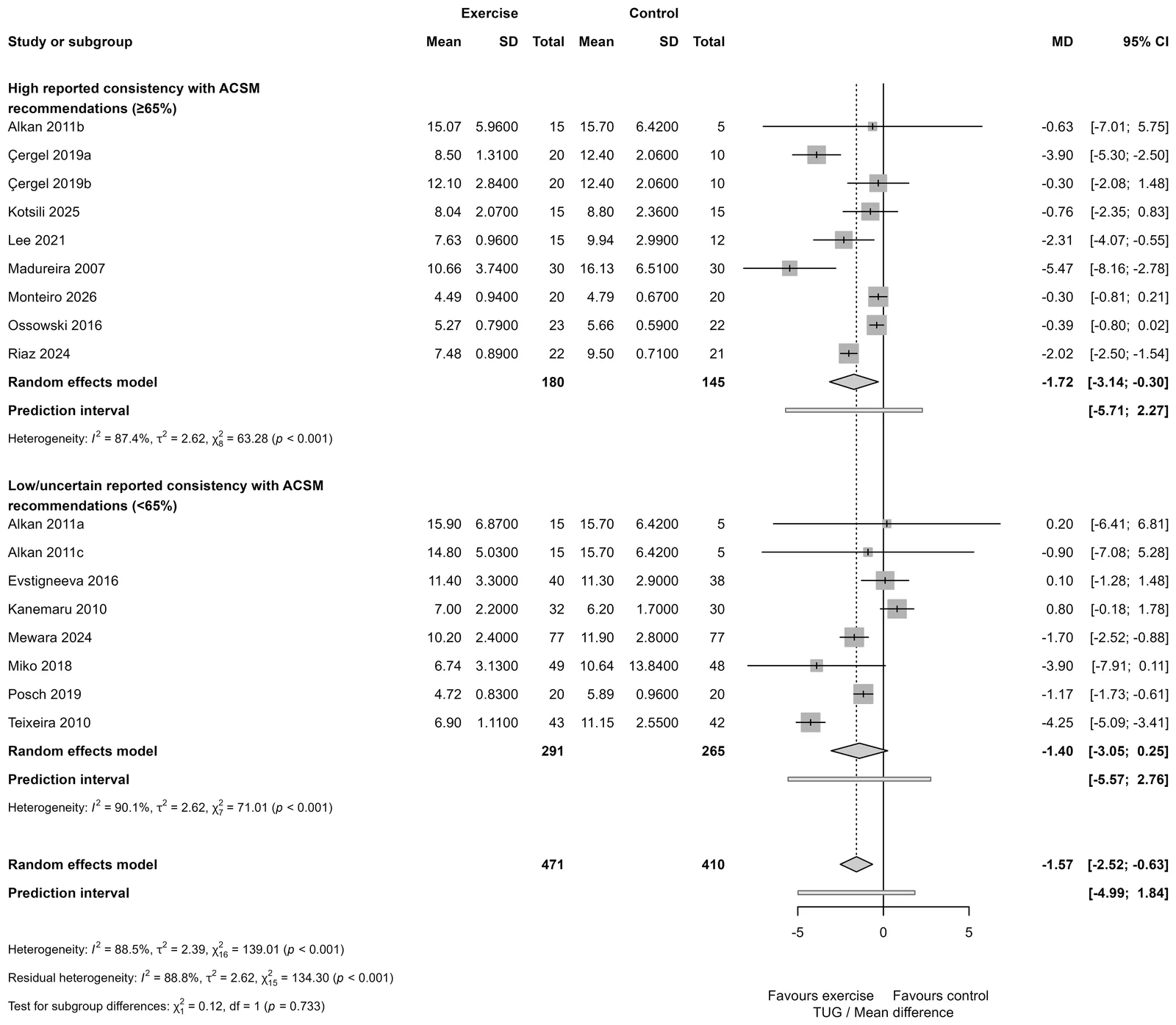
TUG results grouped by reported consistency with ACSM recommendations (primary 65% threshold).

Exercise reduced TUG time (17 effect estimates; MD = −1.57 s, 95% CI −2.52 to −0.63; p = 0.003; τ² = 2.39; I² = 88.5%; 95% prediction interval −4.99 to 1.84). The MD was −1.72 s (95% CI −3.14 to −0.30) in the high reported-consistency subgroup and −1.40 s (95% CI −3.05 to 0.25) in the low/uncertain subgroup. The formal test for subgroup differences was not statistically significant (p = 0.733; **Figure 4**).

### 3.6 Berg balance scale: overall and reported-consistency subgroup effects

**Figure 5:**
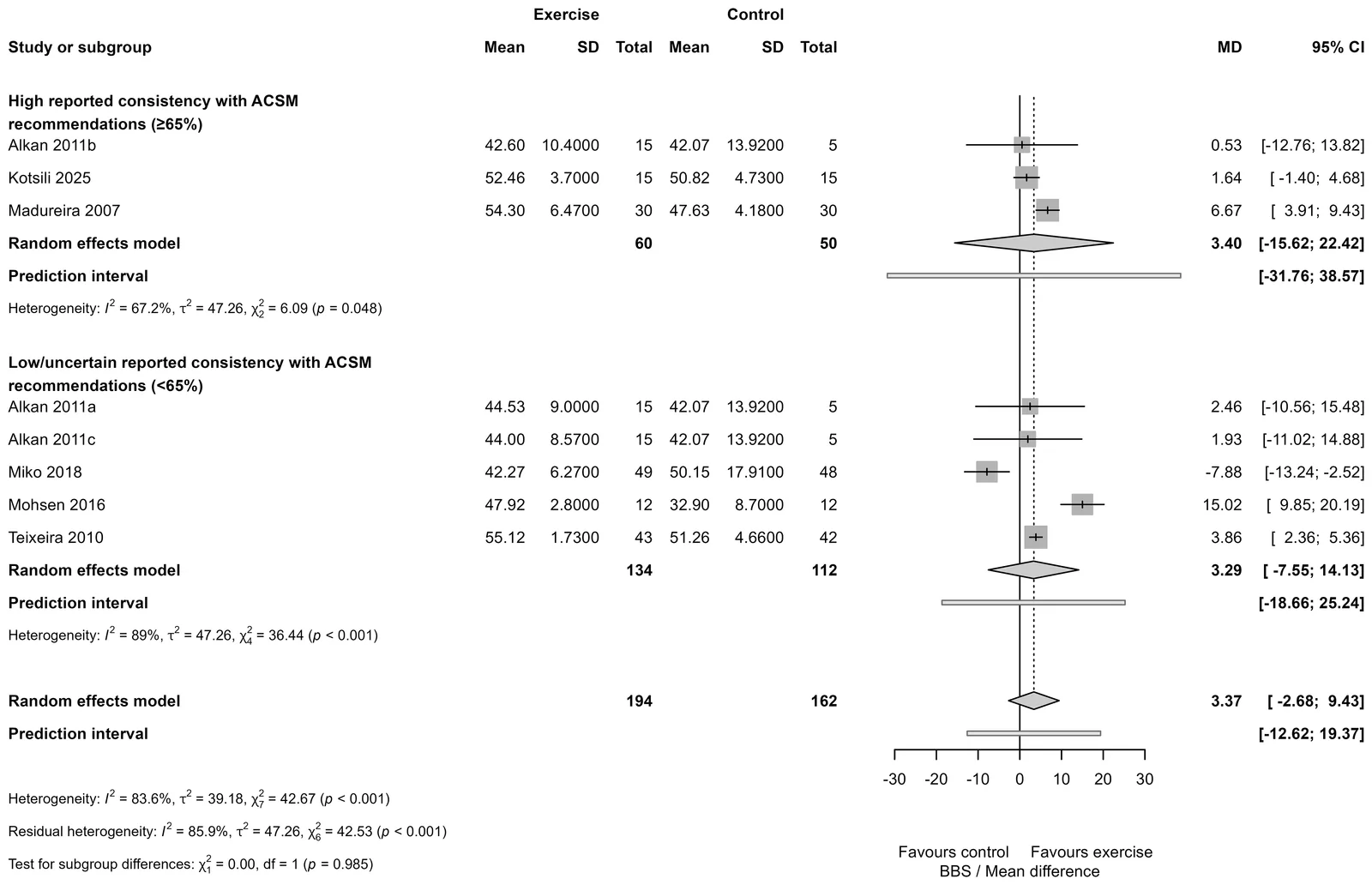
BBS results grouped by reported consistency with ACSM recommendations (primary 65% threshold).

The estimated effect on BBS was highly uncertain (eight effect estimates; MD = 3.37 points, 95% CI −2.68 to 9.43; p = 0.229; τ² = 39.18; I² = 83.6%; 95% prediction interval −12.62 to 19.37). The MD was 3.40 points (95% CI −15.62 to 22.42) in the high reported-consistency subgroup and 3.29 points (95% CI −7.55 to 14.13) in the low/uncertain subgroup. The test for subgroup differences was not statistically significant (p = 0.985; **Figure 5**).

### 3.7 30-second sit-to-stand test: overall and reported-consistency subgroup effects

**Figure 6:**
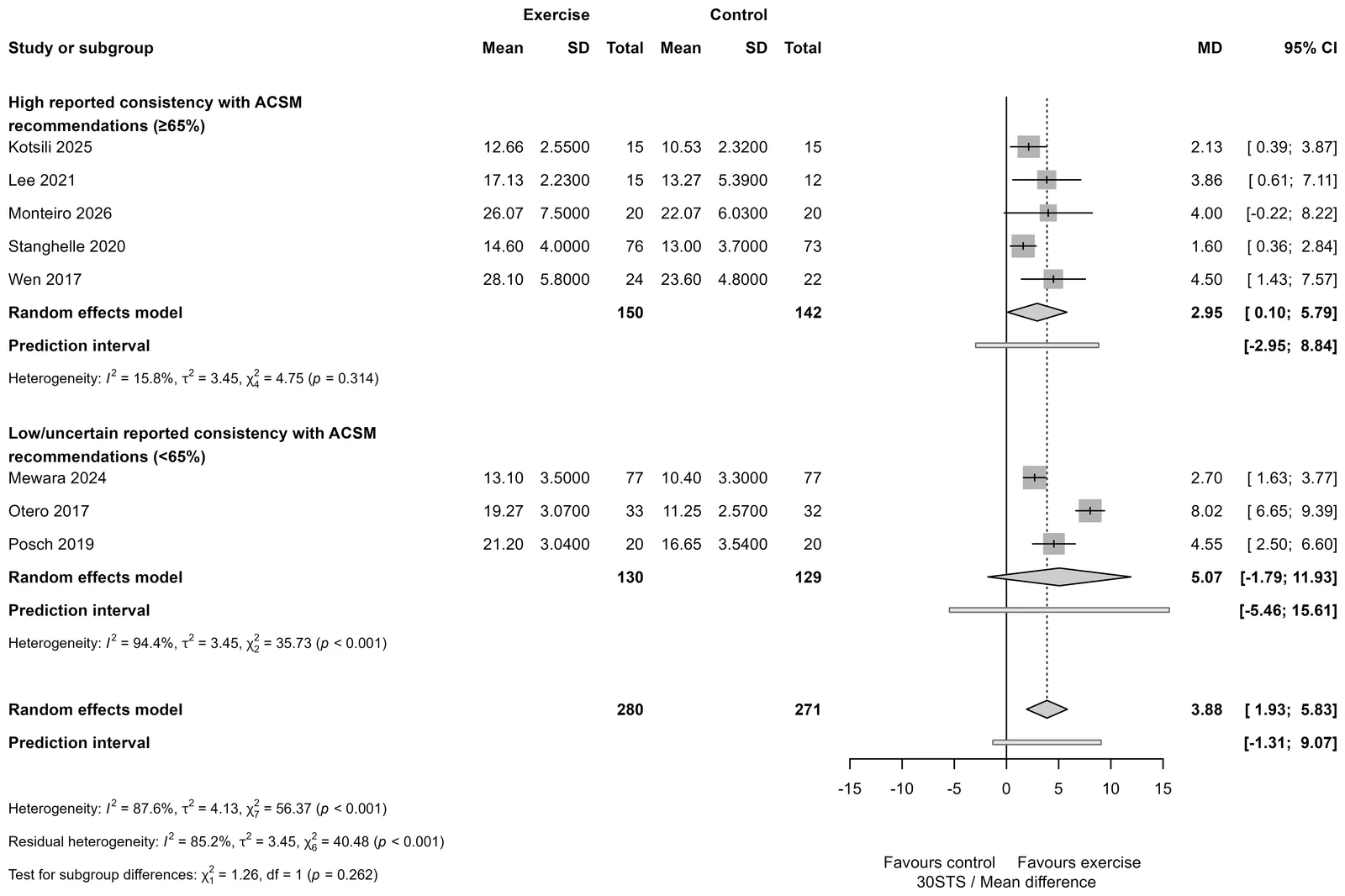
30STS results grouped by reported consistency with ACSM recommendations (primary 65% threshold).

Exercise increased the number of 30STS repetitions (eight effect estimates; MD = 3.88, 95% CI 1.93 to 5.83; p = 0.002; τ² = 4.13; I² = 87.6%; 95% prediction interval −1.31 to 9.07). The MD was 2.95 repetitions (95% CI 0.10 to 5.79) in the high reported-consistency subgroup and 5.07 repetitions (95% CI −1.79 to 11.93) in the low/uncertain subgroup. The test for subgroup differences was not statistically significant (p = 0.262; **Figure 6**).

### 3.8 Sensitivity analyses and meta-regression

Raising the classification threshold to 70% (**Supplementary Table 5**; **Supplementary Figures S1**, **S2**) or assigning U half weight (**Supplementary Table 6**; **Supplementary Figures S3**, **S4**) changed the subgroup allocation of several TUG and BBS intervention arms but did not produce a statistically significant subgroup interaction; no 30STS arm was reclassified under half-weight scoring. In leave-one-study-arm-out analyses (**Supplementary Tables S7**–**S9**; **Supplementary Figures S5**–**S7**), TUG estimates ranged from −1.32 to −1.76 s, remained statistically significant throughout, and had I² values of 82.2%–89.2%. The BBS result was unstable: after excluding Mikó 2018, the pooled estimate was 5.40 points (95% CI 0.34 to 10.46), and I² decreased from 83.6% to 73.9%. The direction of the 30STS result remained stable; after excluding Otero 2017, I² decreased from 87.6% to 31.5%, and the MD was 2.87 repetitions (95% CI 1.70 to 4.04). Analyses excluding studies at outcome-specific high risk of bias under RoB 2 are shown in **Supplementary Figures S8**, **S9**. After these exclusions, the TUG estimate still favoured exercise but was attenuated (MD = −1.00 s, 95% CI −1.84 to −0.16; I² = 73.7%), whereas the BBS result remained highly uncertain (MD = 2.44 points, 95% CI −6.85 to 11.72; I² = 86.6%). This sensitivity analysis was not conducted for 30STS because no contributing study was at outcome-specific high risk of bias. Exploratory univariable meta-regression results are reported in **Supplementary Tables S10**-**S12** and **Supplementary Figures S13**-**S15**. Mean age, intervention duration, and weekly frequency were not statistically associated with TUG or BBS effects. For 30STS, however, higher mean age was associated with a smaller positive mean difference (β = −0.31 repetitions/year, 95% CI −0.53 to −0.09; p = 0.016; k = 7), while intervention duration, weekly frequency, and session duration were not significant. Given the small k, missing moderator data, and multiple exploratory tests, these findings do not establish causal effect modifiers.

### 3.9 Small-study effects and certainty of evidence

Funnel plots and asymmetry tests are presented in **Supplementary Figures S10**-**S12**. There was no statistically significant evidence of small-study effects: Egger and Begg–Mazumdar P values were 0.3969 and 0.7150 for TUG, 0.8372 and 0.7195 for BBS, and 0.7260 and 0.5484 for 30STS, respectively. However, the BBS and 30STS tests were each based on only eight effect estimates and were therefore underpowered; non-significant asymmetry tests should not be interpreted as evidence that publication bias is absent. GRADE certainty is summarised in **Table 2**: certainty was low for TUG and 30STS and very low for BBS.

**Table 2:** GRADE assessment of the certainty of evidence for TUG, BBS, and 30STS.

Outcome	K	Study design	Risk of bias	Inconsistency	Indirectness	Imprecision	Other considerations	N(E/C)	MD [95% CI]	Certainty	Importance
TUG	17	RCT	serious	serious	not serious	not serious	none	471/410	-1.57 [-2.52, -0.63]	⨁⨁◯◯ Low	CRITICAL
BBS	8	RCT	serious	serious	not serious	serious	none	194/162	3.37 [-2.68, 9.43]	⨁◯◯◯ Very low	CRITICAL
30STS	8	RCT	serious	serious	not serious	not serious	none	280/271	3.88 [1.93, 5.83]	⨁⨁◯◯ Low	CRITICAL

k, number of effect estimates; N(E/C), sample size in exercise/control groups; MD, mean difference; CI, confidence interval; E, exercise intervention; C, control group; RCT, randomised controlled trial; TUG, Timed Up and Go test; BBS, Berg Balance Scale; 30STS, 30-second sit-to-stand test.

## 4 Discussion

This review evaluated the effects of exercise on immediate post-intervention fall-related functional performance in postmenopausal women with osteoporosis or low bone mass and explored whether effects differed according to the reported consistency of exercise prescriptions with ACSM recommendations. Overall, exercise improved mean TUG and 30STS performance, whereas the effect on BBS remained highly uncertain. Formal tests for subgroup differences were not statistically significant for any outcome. Thus, the findings support the possibility that exercise improves functional mobility and repeated sit-to-stand performance, but the available evidence does not demonstrate superior effects for exercise prescriptions with higher reported consistency with ACSM recommendations.

Exercise reduced TUG time by a mean of 1.57 s and increased 30STS performance by a mean of 3.88 repetitions. The 95% confidence intervals for both overall effects did not cross the null, indicating statistically significant mean effects. In particular, the increase in 30STS repetitions reflects improved lower-limb functional strength and repeated sit-to-stand capacity and should not be described merely as a favourable trend. However, no clinical-importance threshold applies uniformly across the diverse populations with osteoporosis or low bone mass included here, so it remains uncertain whether these mean changes consistently represent clinically important improvement. Prediction intervals for both TUG and 30STS crossed the null, indicating that effects may vary substantially across future exercise programmes. The wide BBS confidence interval included both no effect and potentially meaningful benefit; together with very-low-certainty GRADE evidence, this result is better described as highly uncertain rather than merely non-significant. Overall, the TUG and 30STS findings were supported by low-certainty evidence and the BBS finding by very-low-certainty evidence, precluding firm clinical conclusions.

The analysis of reported consistency with ACSM recommendations was exploratory. Statistical significance in one subgroup but not another does not establish a difference between subgroups, and the formal subgroup tests were not statistically significant for any of the three outcomes. Raising the classification threshold from 65% to 70% and assigning half weight to unclear or unreported components reclassified several TUG and BBS intervention arms but did not materially change the conclusion concerning subgroup differences. This offers some support for consistency across scoring assumptions while also showing that arm allocation depends on the scoring rules. Importantly, the component weights, operational exercise domains, and 65% threshold were researcher-defined rather than ACSM-specified or validated. Excluding domains that were not included in a programme from the total-score denominator may also make programmes covering fewer domains more likely to receive a high score. Although unclear or unreported components and components clearly not meeting the criteria were coded separately as U and N, both received zero in the primary analysis. The measure therefore reflects how the exercise prescription reported in the publication corresponds to ACSM recommendations; it cannot substitute for assessment of actual intervention delivery, supervision quality, or participant adherence.

The overall direction of the TUG and 30STS findings is broadly consistent with previous systematic reviews. Resistance, balance, weight-bearing, and multicomponent exercise may improve physical function and fall-related capacity in older or osteoporotic populations (Howe et al., 2011; Gillespie et al., 2012; Giangregorio et al., 2014, 2015; Sherrington et al., 2019). TUG integrates sequential standing, walking, turning, and sitting, whereas 30STS reflects repeated lower-limb force production and functional endurance; both may therefore respond to resistance training, stepping, weight-bearing activity, and multicomponent exercise. By contrast, BBS covers a broader range of static and dynamic balance tasks, and its response may depend more strongly on baseline balance limitation, task specificity, progression, supervision, and programme duration. High between-study heterogeneity and instability in the BBS leave-one-out analysis indicate that a single pooled effect cannot fully represent differences among exercise programmes and study populations. Included studies also differed in age, osteoporosis or low-bone-mass status, history of vertebral fracture, baseline function, and intervention design, all of which may influence exercise tolerance and scope for improvement. Exploratory meta-regression did not identify stable effect modifiers. The association between mean age and 30STS effect size was based on only seven estimates and may reflect small-sample variation, multiple testing, or study-level confounding; non-significant findings for other moderators do not establish that those factors are unrelated to exercise effects. Beyond exercise type, frequency, intensity, and duration, instruction and motor-learning strategies may also influence functional outcomes. It has been suggested that internal or external attentional focus and motor-learning strategies may affect exercise-related functional performance (Kuzu et al., 2025). Instruction, feedback, engagement, progression, and supervision may therefore interact with exercise type and dose. Future trials should report these intervention-delivery features more completely so that the effects of the prescribed exercise can be distinguished from the quality of programme delivery.

Finally, TUG, BBS, and 30STS are fall-related functional tests rather than direct measures of actual falls, fracture risk, or fall incidence. The present findings therefore cannot be interpreted as evidence that exercise directly reduces falls or fractures. Adverse events and safety outcomes were reported inconsistently across the included studies and were not quantitatively pooled. Particularly for women with vertebral fractures or severe osteoporosis, resistance training, impact activities, and highly challenging balance training should be selected according to individual circumstances, with appropriate progression and supervision. Future randomised trials should report functional performance, actual falls, fractures, adverse events, exercise adherence, and long-term follow-up concurrently to clarify the clinical benefits and safety boundaries of exercise interventions.

## 5 Strengths and limitations

### 5.1 Strengths

This study has several strengths. First, it focused on postmenopausal women with osteoporosis or low bone mass, a clinically well-defined population. Only randomised controlled trials were included, and TUG, BBS, and 30STS were used to assess functional mobility, balance, and lower-limb functional strength, thereby capturing several dimensions of fall-related functional performance. Second, alongside the overall effects of exercise, we explored the relation between reported exercise-prescription components and ACSM recommendations. Third, outcome-specific RoB 2 and GRADE assessments were combined with formal tests for subgroup differences, Hartung–Knapp inference, prediction intervals, and multiple sensitivity analyses to examine the robustness of the main findings under alternative analytic assumptions.

## 6 Limitations

This study also has limitations. The evidence base was limited, and the BBS and 30STS analyses each included only eight effect estimates, leaving subgroup, sensitivity, and publication-bias analyses underpowered. Between-study heterogeneity was high and may reflect differences in participant age, osteoporosis or low-bone-mass status, fracture history, baseline function, exercise type, training dose, supervision, and comparator conditions. In addition, because the meta-analyses were based on post-intervention means rather than change scores or adjusted estimates, residual baseline imbalances—particularly in small trials—may have influenced the pooled effect estimates. The score for reported consistency with ACSM recommendations was researcher-developed; its component weights and classification threshold have not been externally validated, and the score depends on the completeness of exercise-prescription reporting in the original studies. Furthermore, 10 reports could not be retrieved despite the retrieval attempts described in the **Supplementary Material**. Their non-retrieval may have introduced selection bias and reduced the completeness of the available evidence. No language restrictions were applied; however, eligibility was restricted to full-text articles published in peer-reviewed journals. Clinical trial registries and grey-literature sources were not searched separately, and reference lists were not systematically searched. Consequently, unpublished, ongoing, or poorly indexed studies may have been missed.

## 7 Conclusion

Exercise may improve immediate post-intervention TUG and 30STS performance in postmenopausal women with osteoporosis or low bone mass, but heterogeneity was high and certainty of evidence was low. Evidence for BBS remains very uncertain. No clear difference was found between the high and low/uncertain groups for reported consistency with ACSM recommendations; the classification analysis should therefore be interpreted as exploratory rather than as evidence that one prescription category is superior.

## Data Availability

The original contributions presented in the study are included in the article/**Supplementary Material**. Further inquiries can be directed to the corresponding author.
