# Supplementary material for: Effects of exercise interventions on fall-related functional performance in postmenopausal women with osteoporosis or low bone mass: a systematic review and meta-analysis with an exploratory analysis of reported consistency with ACSM recommendations
Source: Front Physiol. 2026 Aug 12;17:1903705. doi: 10.3389/fphys.2026.1903705 (PMC13506822; doi:10.3389/fphys.2026.1903705)
Supplement: Supplementary file 1 [file SupplementaryFile1.pdf]

Supplementary Material

1. Search strategy

The exact database-specific search strategies used for this review are presented below. Six electronic databases were searched from inception to 3 May 2026: PubMed, Embase, Web of Science, Scopus, the Cochrane Library, and CINAHL via EBSCO. Controlled vocabulary and free-text terms covered osteoporosis or low bone mass, postmenopausal women, exercise, and randomised or controlled clinical trials.

After title and abstract screening, 83 reports were sought for retrieval. Ten reports could not be retrieved, leaving 73 full-text reports for eligibility assessment. Of these, 55 reports were excluded for the reasons presented in Table S2, and 18 reports representing 18 independent studies were included in the systematic review. Attempts to obtain reports that were not immediately accessible included contacting the corresponding authors by email to request the full texts. Ten reports remained unavailable after these attempts and are identified in Table S2.

Table S1. Search strategies and database yields across six databases

Database	Search strategy	Amount
	**PubMed**	
#1	Search: (Osteoporosis[MeSH Terms]) OR (Osteoporosis, Postmenopausal[MeSH Terms]) OR (Osteopenia[MeSH Terms])	24995
#2	Search: (osteoporosis[Title/Abstract]) OR (osteopenia[Title/Abstract]) OR ("low bone mass"[Title/Abstract]) OR ("low bone density"[Title/Abstract]) OR ("bone loss"[Title/Abstract]) OR ("reduced bone mass"[Title/Abstract])	36500
#3	#1 OR #2	43596
#4	Search:(Exercise[MeSH Terms]) OR (Exercise Therapy[MeSH Terms]) OR (Motor Activity[MeSH Terms])	105915
#5	Search:(exercise[Title/Abstract]) OR (training[Title/Abstract]) OR ("physical training"[Title/Abstract]) OR ("resistance training"[Title/Abstract]) OR ("strength training"[Title/Abstract]) OR ("aerobic training"[Title/Abstract]) OR ("balance training"[Title/Abstract]) OR ("physical activity"[Title/Abstract]) OR (walking[Title/Abstract]) OR	252391

	("tai chi"[Title/Abstract]) OR (yoga[Title/Abstract])	
#6	#4 OR #5	271339
#7	Search:(Randomized Controlled Trial[Publication Type]) OR (randomized[Title/Abstract]) OR (randomised[Title/Abstract]) OR (randomly[Title/Abstract]) OR (trial[Title/Abstract])	1026237
#8	Search: (Postmenopause[MeSH Terms]) OR (Women[MeSH Terms])	14400
#9	Search:(postmenopausal[Title/Abstract]) OR (postmenopause[Title/Abstract]) OR ("post-menopausal"[Title/Abstract]) OR (women[Title/Abstract]) OR (woman[Title/Abstract]) OR (female[Title/Abstract])	339845
#10	Search:#8 OR #9	340858
#11	Search: #3 AND #6 AND #7 AND #10	603
	**Web of Science**	
#1	TS=(osteoporosis OR osteopenia OR "low bone mass" OR "low bone density" OR "bone loss" OR "reduced bone mass" OR "postmenopausal osteoporosis")	176431
#2	TS=(exercise OR training OR "physical training" OR "resistance training" OR "strength training" OR "aerobic training" OR "balance training" OR "physical activity" OR walking OR "tai chi" OR yoga)	16068
#3	TS=(postmenopausal OR postmenopause OR "post-menopausal" OR women OR woman OR female)	9189
#4	TS=(randomized OR randomised OR randomly OR trial)	1165344
#5	#1 AND #2 AND #3 AND #4	2294
	**Embase**	
#1	Search: 'osteoporosis'/exp OR 'postmenopausal osteoporosis'/exp OR 'osteopenia'/exp	246495
#2	Search: osteoporosis:ti,ab OR osteopenia:ti,ab OR "low bone mass":ti,ab OR "low bone density":ti,ab OR	195242

	"bone loss":ti,ab OR<br>"reduced bone mass":ti,ab	
#3	#1 OR #2	281030
#4	Search: 'exercise'/exp OR 'exercise therapy'/exp	640407
#5	Search: exercise:ti,ab OR training:ti,ab OR<br>"physical training":ti,ab OR<br>"resistance training":ti,ab OR<br>"strength training":ti,ab OR<br>"aerobic training":ti,ab OR<br>"balance training":ti,ab OR<br>"physical activity":ti,ab OR<br>walking:ti,ab OR<br>"tai chi":ti,ab OR<br>yoga:ti,ab	1695173
#6	#4 OR #5	1873651
#7	Search: 'randomized controlled trial'/exp OR random:ti,ab OR trial:ti,ab	2604687
#8	Search: 'postmenopause'/exp OR 'woman'/exp	14659860
#9	Search: postmenopausal:ti,ab OR<br>postmenopause:ti,ab OR<br>"post-menopausal":ti,ab OR<br>women:ti,ab OR<br>woman:ti,ab OR<br>female:ti,ab	3785686
#10	#8 OR #9	15044709
#11	#3 AND #6 AND #7 AND #10	2332
	**Cochrane**	
#1	(Osteoporosis):ti,ab,kw OR<br>(Postmenopausal Osteoporosis):ti,ab,kw<br>OR (Osteopenia):ti,ab,kw	13672
#2	(osteoporosis):ti,ab,kw OR<br>(osteopenia):ti,ab,kw OR<br>("low bone mass"):ti,ab,kw OR ("low bone density"):ti,ab,kw OR<br>("bone loss"):ti,ab,kw OR ("reduced bone mass"):ti,ab,kw	19062
#3	#1 OR #2	19062
#4	(Exercise):ti,ab,kw OR (Exercise Therapy):ti,ab,kw	154608
#5	(exercise):ti,ab,kw OR (training):ti,ab,kw OR<br>("physical training"):ti,ab,kw OR	295473

	("resistance training"):ti,ab,kw OR ("strength training"):ti,ab,kw OR ("aerobic training"):ti,ab,kw OR ("balance training"):ti,ab,kw OR ("physical activity"):ti,ab,kw OR (walking):ti,ab,kw OR ("tai chi"):ti,ab,kw OR (yoga):ti,ab,kw	
#6	#4 OR #5	295473
#7	(Randomized Controlled Trial):ti,ab,kw OR (random):ti,ab,kw OR (trial):ti,ab,kw OR (controlled):ti,ab,kw	1423882
#8	(postmenopausal):ti,ab,kw OR (postmenopause):ti,ab,kw OR ("post-menopausal"):ti,ab,kw OR (women):ti,ab,kw OR (woman):ti,ab,kw OR (female):ti,ab,kw	1118104
#9	#3 AND #6 AND #7 AND #8	1267
	**Scopus**	
#1	TITLE-ABS-KEY (osteoporosis OR osteopenia OR "low bone mass" OR "low bone density" OR "bone loss" OR "reduced bone mass" OR "postmenopausal osteoporosis")	11120
#2	TITLE-ABS-KEY (exercise OR training OR "physical training" OR "resistance training" OR "strength training" OR "aerobic training" OR "balance training" OR "physical activity" OR walking OR "tai chi" OR yoga)	3077120
#3	TITLE-ABS-KEY (postmenopausal OR postmenopause OR "post-menopausal" OR women OR woman OR female)	91045
#4	TITLE-ABS-KEY (randomized OR randomised OR randomly OR trial)	4141146
#5	#1 AND #2 AND #3 AND #4	2958
	**CINAHL (EBSCO)**	
#1	(MH "Osteoporosis") OR (MH "Osteoporosis, Postmenopausal") OR (MH "Osteopenia")	25610
#2	TI osteoporosis OR AB osteoporosis OR TI	16092

	osteopenia OR AB osteopenia OR<br>TI "low bone mass" OR AB "low bone mass" OR<br>TI "low bone density" OR AB "low bone density" OR<br>TI "bone loss" OR AB "bone loss" OR<br>TI "reduced bone mass" OR AB "reduced bone mass"	
#3	#1 OR #2	30014
#4	(MH "Exercise") OR (MH "Exercise Therapy")	142861
#5	TI exercise OR AB exercise OR TI training OR AB training OR<br>TI "physical training" OR AB "physical training" OR<br>TI "resistance training" OR AB "resistance training" OR<br>TI "strength training" OR AB "strength training" OR<br>TI "aerobic training" OR AB "aerobic training" OR<br>TI "balance training" OR AB "balance training" OR<br>TI "physical activity" OR AB "physical activity" OR<br>TI walking OR AB walking OR<br>TI "tai chi" OR AB "tai chi" OR<br>TI yoga OR AB yoga	447475
#6	#4 OR #5	510772
#7	(MH "Randomized Controlled Trials") OR TI random OR<br>AB random OR TI trial OR AB trial	717905
#8	(MH "Postmenopause") OR (MH "Women+") OR<br>TI postmenopausal OR AB postmenopausal OR<br>TI postmenopause OR AB postmenopause OR<br>TI "post-menopausal" OR AB "post-menopausal" OR<br>TI women OR AB women OR	6001

	TI woman OR AB woman OR TI female OR AB female	
#9	#3 AND #6 AND #7 AND #8	669
Total	PubMed+Embase+Web of Science+Cochrane+Scopus+CINAHL (EBSCO)	10,123

Table.S2 Full-text articles excluded with reasons

**Author, year**	**Study title**	**Reason for exclusion**
Wayne, 2012	Impact of Tai Chi exercise on multiple fracture-related risk factors in post-menopausal osteopenic women: a pilot pragmatic, randomized trial	Outcome or data unavailable
Nawrat-Szołtysik, 2018	Effect of physical activity on the sequelae of osteoporosis in female residents of residential care facilities	Intervention or control mismatch
Altaş, 2026	Investigating the Effects of Face-to-face Rehabilitation and Telerehabilitation on Muscle Strength, Balance, and Quality of Life in Postmenopausal Osteoporosis Patients: Randomized Controlled Trial	Intervention or control mismatch
Marchesei, 2012	Effects of a weight-bearing exercise training on bone mineral density and neuromuscular function of osteopenic women	Outcome or data unavailable
Arnold, 2008	A randomized clinical trial of aquatic versus land exercise to improve balance, function, and quality of life in older women with osteoporosis	Outcome or data unavailable
Atashak, 2026	Effects of high-intensity interval training and concurrent training on body composition and metabolic profile in postmenopausal women with overweight or obesity: a clinical trial	Population mismatch
Aveiro, 2017	Water- versus land-based treatment for postural control in postmenopausal osteoporotic women: a randomized, controlled trial	Outcome or data unavailable
Barker, 2019	Physiotherapy rehabilitation for osteoporotic vertebral fracture—a randomised controlled trial and economic evaluation (PROVE trial)	Population mismatch

Makarova, 2020	Improvement of core muscle strength after the physical rehabilitation complex in patients with osteoporotic vertebral fractures	Full text unavailable
Bergland, 2010	Effect of exercise on mobility, balance, and health-related quality of life in osteoporotic women with a history of vertebral fracture: a randomized, controlled trial	Outcome or data unavailable
Cao, 2019	Exercise Training at Maximal Fat Oxidation Intensity for Overweight or Obese Older Women: A Randomized Study	Population mismatch
Carter, 2001	Results of a 10 week community based strength and balance training programme to reduce fall risk factors: a randomised controlled trial in 65–75 year old women with osteoporosis	Duplicate or secondary report
Carter, 2002	Community-based exercise program reduces risk factors for falls in 65- to 75-year-old women with osteoporosis: randomized controlled trial	Outcome or data unavailable
Conradsson, 2019	The effects of dual-task balance training on gait in older women with osteoporosis: A randomized controlled trial	Outcome or data unavailable
Cunha, 2018	Resistance training performed with single-set is sufficient to reduce cardiovascular risk factors in untrained older women: The randomized clinical trial. Active Aging Longitudinal Study	Population mismatch
Cunha, 2021	Comparison of low and high volume of resistance training on body fat and blood biomarkers in untrained older women: A randomized clinical trial	Population mismatch
Daca, 2023	Biopsychosocial Effects of a Conventional Exercise Program and Culturally Relevant Activities in Older Women From Mozambique	Population mismatch
Watson, 2015	High intensity progressive resistance training for postmenopausal women with low bone mass: Early findings from the LIFTMOR trial	Full text unavailable
Deniz, 2025	The effect of aerobic exercise on bone formation and resorption markers and the quality of life tests in postmenopausal osteopenic patients	Outcome or data unavailable
Devereux,	Effects of a water-based program on women 65 years and over: A randomised controlled trial	Outcome or data unavailable

2005		
Dizdar, 2017	Effects of Balance-Coordination, Strengthening and Aerobic Exercises to Prevent Falls in Postmenopausal Patients With Osteoporosis: A 6-Month Randomized Parallel Prospective Study	Outcome or data unavailable
Dohrn, 2017	Short- and Long-Term Effects of Balance Training on Physical Activity in Older Adults With Osteoporosis: A Randomized Controlled Trial	Outcome or data unavailable
Bergland, 2010	Efficacy of exercise regarding improving balance, mobility and health-related quality of life among elderly women with osteoporosis	Full text unavailable
EL-HADIDY, 2025	Effect of training using the Biodex system on dynamic balance and physical performance: a randomized controlled balance-training trial in hemodialysis patients with osteopenia	Population mismatch
Eladl, 2025	Progressive resisted exercise program combined with aerobic exercise on osteoporotic systemic lupus erythematous patients: a prospective randomized controlled trial	Population mismatch
Fleisher, 2011	Effects of Weight-Bearing and Resistance Exercises on Lower Extremity Strength, Postural Stability, and Quality of Life in Postmenopausal Women With Low Bone Mass	Outcome or data unavailable
Patru, 2017	Effects of tai chi exercise on muscle strength and balance in postmenopausal women with osteopaenia	Full text unavailable
Forsyth, 2023	Menopause Osteoporosis and Bone Intervention Using Lifestyle Exercise: A Randomized Controlled Study	Outcome or data unavailable
Ordahan, 2018	Osteoporozlu Hastalarda Denge Cihazı ile Yapılan Denge Egzersizlerinin Yaşam Kalitesi ve Denge Üzerine Etkisi: randomize Klinik Çalışma	Full text unavailable
Bergland, 2012	Effect of exercise on falls efficacy in osteoporotic women with a history of vertebral fracture: a randomized, controlled trial	Full text unavailable
Gómez-Tomás, 2018	Effect of a 1-year elastic band resistance exercise program on cardiovascular risk profile in postmenopausal women	Population mismatch

Halvarsson, 2015	Balance training with multi-task exercises improves fall-related self-efficacy, gait, balance performance and physical function in older adults with osteoporosis: a randomized controlled trial	Population mismatch
Halvarsson, 2016	Long-term effects of a progressive and specific balance-training programme with multi-task exercises for older adults with osteoporosis: A randomized controlled study	Population mismatch
Hourigan, 2008	Positive effects of exercise on falls and fracture risk in osteopenic women	Population mismatch
Khan, 2019	Effects of osteoanabolic exercises on bone mineral density of osteoporotic females: A randomized controlled trial	Outcome or data unavailable
Gibbs, 2018	Effect of home exercise on functional performance, posture, quality of life and pain in older women with vertebral fractures: a pilot feasibility trial	Full text unavailable
Khan, 2023	Effects of osteoanabolic exercises on gait, balance and fear of fall among osteoporotic females	Intervention or control mismatch
Khoo, 2019	The intense musculoskeletal, functional performance and cardiometabolic training (Impact) exercise intervention in postmenopausal Singaporean women: a pilot study assessing feasibility, safety and acceptability	Full text unavailable
Küçükçakır, 2012	Effects of Pilates exercises on pain, functional status and quality of life in women with postmenopausal osteoporosis	Outcome or data unavailable
Liu, 2015	The Effect of the Modified Eighth Section of Eight-Section Brocade on Osteoporosis in Postmenopausal Women: A Prospective Randomized Trial	Intervention or control mismatch
Liu-Ambrose, 2012	Both Resistance and Agility Training Reduce Fall Risk in 75–85 Year Old Women with Low Bone Mass: A Six-Month Randomized Controlled Trial	Outcome or data unavailable
Lyu, 2025	Effects of Tailored Mindful Exercise on Back Pain, Balance, Kinesiophobia, Anxiety and Depression in Older Patients with Primary Osteoporosis: A Randomized Controlled Trial	Population mismatch

Marques, 2009	Effects of resistance and multicomponent exercise on lipid profiles of older women	Population mismatch
Mawi, 2009	Effect of aerobic exercise on blood lipid levels in postmenopausal women	Population mismatch
Mitchell, 1998	Physiological Effects of Exercise on Post-menopausal Osteoporotic Women	Outcome or data unavailable
Mohanka, 2006	Serum Lipoproteins in Overweight/Obese Postmenopausal Women: A One-Year Exercise Trial	Population mismatch
Morone, 2016	Wii Fit is effective in women with bone loss condition associated with balance disorders: a randomized controlled trial	Intervention or control mismatch
Motalebi, 2019	Effect of a Physical Activity Program on Serum Biochemical Parameters among the Elderly Women	Population mismatch
Barker, 2018	Physiotherapy rehabilitation for osteoporotic vertebral fracture: a randomised controlled trial and economic evaluation (PROVE trial): ISRCTN 49117867	Full text unavailable
Nawrat-Szołtysik, 2019	Effect of physical activity on the quality of life in osteoporotic females living in residential facilities: A Randomized Controlled Trial	Outcome or data unavailable
Nieman, 1993	Physical Activity and Serum Lipids and Lipoproteins in Elderly Women	Population mismatch
Oksuz, 2017	The effect of the clinical pilates exercises on kinesiophobia and other symptoms related to osteoporosis: Randomised controlled trial	Outcome or data unavailable
Oliveira, 2019	Effect of circuit resistance training on blood biomarkers of cardiovascular disease risk in older women	Population mismatch
Traistaru, 2019	Low back pain in females with severe osteoporosis: assessment and rehabilitation	Full text unavailable
Olsen, 2014	The effect of exercise and education on fear of falling in elderly women with osteoporosis and a history of vertebral fracture: results of a randomized controlled trial	Outcome or data unavailable
Papaioannou, 2003	Efficacy of home-based exercise for improving quality of life among elderly women with symptomatic osteoporosis-related vertebral fractures	Outcome or data unavailable

Ramos, 2019	A single whole body vibration session influences quadriceps muscle strength, functional mobility and balance of elderly with osteopenia and/or osteoporosis? Pragmatic clinical trial	Population mismatch
Rezaei, 2023	The effect of six week virtual reality training on the improvement of functional balance in women with type-I osteoporosis: A preliminary study	Intervention or control mismatch
Saito, 2021	Effect of eldecalcitol on muscle function and fall prevention in Japanese postmenopausal women: A randomized controlled trial	Intervention or control mismatch
Stojanović, 2021	Effects of Chair-Based, Low–Load Elastic Band Resistance Training on Functional Fitness and Metabolic Biomarkers in Older Women	Population mismatch
Stolzenberg, 2013	Vibration or Balance Training on Neuromuscular Performance in Osteopenic Women	Intervention or control mismatch
Tantanasest, 2026	Comparative Effects of Weighted Vest and Whole-Body Vibration Training on Bone and Muscle Health in Osteopenia	Intervention or control mismatch
Tomeleri, 2023	Effect of Resistance Exercise Order on Cardiovascular Disease Risk Factors in Older Women: A Randomized Controlled Trial	Population mismatch
Watson, 2015	Heavy resistance training is safe and improves bone, function, and stature in postmenopausal women with low to very low bone mass: novel early findings from the LIFTMOR trial	Intervention or control mismatch
Zhang, 2022	Effect of a home-based resistance exercise program in elderly participants with osteoporosis: a randomized controlled trial	Population mismatch

2. Characteristics of the included studies

This section summarises the design, population, intervention, comparator, and outcome characteristics of the included randomised controlled trials.

Table S3. Characteristics of the included studies

Author	Country	Year	Population	Age (mean ± SD)	Total	Intervention	Control	Outcome
Alkan et al	Turkey	2011	Osteoporotic women over 65 years	T: 71.13(3.40)<br>C: 70.80(5.02)	T: 15<br>C: 15	Home-based vestibular exercise<br>Length: 4 weeks<br>Freq: daily<br>Duration: 10 reps/day	CON/education	BBS<br>TUG
Çergel et al	Turkey	2019	Postmenopausal women with osteoporosis and vertebral fracture	T: 58.90(4.70)<br>C: 59.65(6.45)	T: 20<br>C: 20	Supervised back extensor strengthening exercise<br>Length: 6 weeks<br>Freq: 3 times/week<br>Duration: NR	CON / usual habits	TUG
Evstigneeva et al	Russia	2016	Postmenopausal women with osteoporosis and vertebral fracture	T: 70.7(8.1)<br>C: 67.6(7.0)	T: 40<br>C: 38	Structured physical exercise<br>Length: 12 months<br>Freq: 2 times/week<br>Duration: 40 min	CON / usual habits	TUG

Author	Country	Year	Population	Age (mean ± SD)	Total	Intervention	Control	Outcome
Kanemaru et al	Japan	2010	Osteoporotic women over 60 years	T: 74.7(6.7) C: 74.3(7.0)	T: 37 C: 32	Home-based daily muscle training Length: 12 months Freq: daily Duration: NR	CON / usual habits	TUG
Kotsili et al	Greece	2025	Postmenopausal women with osteopenia	T: 67.86(5.30) C: 66.73(5.18)	T: 15 C: 15	Multicomponent exercise Length: 16 weeks Freq: 3 times/week Duration: 45 min	CON / usual habits	TUG 30STS BBS
Lee et al	Taiwan	2021	Postmenopausal osteopenic women with osteosarcopenic adiposity	T: 70.13(4.41) C: 71.82(5.23)	T: 15 C: 12	Progressive elastic band resistance exercise Length: 12 weeks Freq: 3 times/week Duration: 55 min	CON/education	TUG 30STS
Madureira et al	Brazil	2007	Older women with osteoporosis	T: 74.57(4.82) C: 73.40(4.61)	T: 30 C: 30	Balance training program Length: 12 months Freq: 1 time/week + home >=3 times/week Duration: 60 min	CON/usual therapy	BBS TUG

Author	Country	Year	Population	Age (mean ± SD)	Total	Intervention	Control	Outcome
Mewara et al	India	2024	Elderly women with osteopenia or osteoporosis	T: 72.3(5.6)<br>C: 71.8(5.9)	T: 77<br>C: 77	Community-based peer-led exercise program<br>Length: 6 months<br>Freq: NR<br>Duration: NR	CON / standard care and education	TUG<br>30STS
Mikó et al	Hungary	2018	Postmenopausal women with osteoporosis and fracture	T: 69.33(4.56)<br>C: 69.10(5.30)	T: 49<br>C: 48	Sensomotor balance training program<br>Length: 12 months<br>Freq: 3 times/week<br>Duration: 30 min	CON/usual therapy	BBS<br>TUG
Mohsen et al	Egypt	2016	Postmenopausal women with osteopenia	T: 59.1(4.1)<br>C: 58.6(3.9)	T: 12<br>C: 12	Weight-bearing exercise<br>Length: 6 weeks<br>Freq: 5 times/week<br>Duration: NR	CON / usual habits	BBS
Monteiro et al	Portugal	2026	Postmenopausal women with osteoporosis	T: 65-80<br>C: 65-80	T: 20<br>C: 20	MCT + HIIT<br>Length: 8 months<br>Freq: 3 times/week<br>Duration: 50-60 min	CON / usual habits	TUG<br>30STS

Author	Country	Year	Population	Age (mean ± SD)	Total	Intervention	Control	Outcome
Ossowski et al	Poland	2016	Postmenopausal women with Osteoporosis and Osteopenia	T: 69.04(4.81) C: 68.32(4.06)	T: 23 C: 22	Nordic walking training Length: 12 weeks Freq: 3 times/week Duration: 60 min	CON / usual habits	TUG
Otero et al	Spain	2017	Postmenopausal women with osteoporosis	T: 57.4(4.8) C: 58.8(4.5)	T: 33 C: 32	Low-intensity balance + strength exercises Length: 24 weeks Freq: 3 times/week Duration: 60 min	CON / usual habits	30STS
Posch et al	Austria	2019	Older women with osteopenia	T: 69.6(5.3) C: 67.4(6.8)	T: 20 C: 20	Mini-trampoline balance + strength + jumping exercises Length: 12 weeks Freq: 2 times/week Duration: 45-60 min	CON/usual therapy	TUG 30STS
Riaz et al	Pakistan	2024	Postmenopausal women with osteopenia	T: 58.27(5.13) C: 58.00(5.52)	T: 22 C: 21	Kinect-based virtual reality training + daily walking Length: 24 weeks Freq: 3 times/week Duration: 45 min	CON / usual habits	TUG

Author	Country	Year	Population	Age (mean ± SD)	Total	Intervention	Control	Outcome
Stanghelle et al	Norway	2020	Older women with osteoporosis and vertebral fracture	T: 74.7(6.1)<br>C: 73.7(5.6)	T: 76<br>C: 73	Multicomponent resistance + balance exercise<br>Length: 12 weeks<br>Freq: 2 times/week<br>Duration: 60 min	CON / usual habits	30STS
Teixeira et al	Brazil	2010	Sedentary postmenopausal women with osteoporosis	T: 63.1(4.53)<br>C: 62.78(4.87)	T: 50<br>C: 50	Progressive load quadriceps training + proprioception exercises<br>Length: 18 weeks<br>Freq: 2 times/week<br>Duration: NR	CON / usual therapy	BBS<br>TUG
Wen et al	Taiwan	2017	Postmenopausal women with osteopenia	T: 57.5(3.5)<br>C: 58.8(3.2)	T: 24<br>C: 22	Group-based step aerobics exercise<br>Length: 10 weeks<br>Freq: 3 times/week<br>Duration: 90 min	CON / usual habits	30STS

Note. Values are presented as mean (SD) unless otherwise stated. T, intervention group; C, control group; CON, control; NR, not reported; Freq, frequency; BBS, Berg Balance Scale; TUG, Timed Up and Go test; 30STS, 30-second sit-to-stand test.

3. Assessment of reported consistency with ACSM recommendations

The assessment compared the exercise prescriptions reported in each article with the osteoporosis exercise recommendations issued by the American College of Sports Medicine and Exercise is Medicine. It evaluated the reported prescription, not participants' actual adherence, treatment fidelity, supervision quality, or intervention safety.

3.1 Reference framework and operational exercise domains

Reported prescriptions were organised into three operational exercise domains: balance-oriented weight-bearing/aerobic activity, resistance training, and flexibility. The combined balance-oriented weight-bearing/aerobic heading did not require every programme to include all three exercise types. Instead, the applicable ACSM criterion was selected according to the exercise prescription reported by the study.

The operational grouping and component weights were developed by the review authors to permit structured comparison across heterogeneous programmes. They were not direct ACSM scoring recommendations. The prespecified component weights were 30%, 35%, and 35% for frequency, intensity, and duration in the balance-oriented weight-bearing/aerobic domain; 30%, 40%, and 30% for frequency, repetitions, and sets in resistance training; and 40% and 60% for frequency and intensity or hold duration in flexibility training.

3.2 Primary scoring and classification

Two reviewers independently classified each applicable prescription item as M, U, or N. M indicated that sufficient information was reported and the prescription met the corresponding criterion. U indicated that information was unclear or not reported, so consistency could not be determined. N indicated that sufficient information was reported but the prescription did not meet the criterion. Disagreements were resolved through discussion, with a third reviewer available for arbitration.

In the primary analysis, M received the full prespecified component weight, whereas U and N received zero. Each domain score was calculated as the sum of the prespecified weights assigned to exercise-prescription components classified as M. Total reported consistency was calculated as the arithmetic mean of the scores for the exercise domains included in that study arm. Exercise domains not included in a programme were omitted from the denominator. Warm-up or cool-down stretching was not treated as an independent flexibility prescription.

Study arms with a total score of at least 65% were classified as having high reported consistency with ACSM recommendations. Scores below 65% were classified as low or uncertain reported consistency. Because the weights and threshold were researcher-defined, alternative threshold and alternative scoring assumptions were examined in sensitivity analyses.

Table S4. Criteria, decision rules, and primary scoring for reported consistency with ACSM recommendations

Author, year	Balance-oriented weight-bearing/aerobic activity							Resistance training							Flexibility					Total reported-consistency score
	Frequency		Intensity		Duration		Score	Frequency (30%)		Repetitions (40%)		Sets (30%)		Score	Frequency (40%)		Intensity / hold(60%)		Score	
	3–5 d/wk		Moderate–high (balance posture 15–30 s)		20–60 min		30/35/35 (%)	2–3 d/wk		8–12 reps		1–2 sets		30/40/30 (%)	5–7 d/wk		Tightness (30–60 s)		40/60 (%)	
Alkan_2011a	7	N	NR	U	NR	U	0.0%													**0.0%**
Alkan_2011b	3	M	NR	U	20	M	65.0%													**65.0%**
Alkan_2011c	7	N	NR	U	20	M	35.0%													**35.0%**
Çergel_2019a								3	M	8–12	M	3	N	70.0%						**70.0%**

Çergel_2019b								3	M	8–12	M	3	N	70.0%						**70.0%**
Evstigneeva_2016								2	M	NR	U	NR	U	30.0%						**30.0%**
Kanemaru_2010								7	N	NR	U	NR	U	0.0%						**0.0%**
Kotsili_2025	3	M	Low–mod	N	30	M	65.0%	3	M	8–12	M	1–2	M	100.0%						**82.5%**
Lee_2021								3	M	10	M	3	N	70.0%						**70.0%**
Madureira_2007	4	M	Low–mod	N	45	M	65.0%													**65.0%**
Mewara_2024	NR	U	NR	U	NR	U	0.0%	NR	U	NR	U	NR	U	0.0%	NR	U	NR	U	0.0%	**0.0%**
Miko_2018	7	N	NR	U	60	M	35.0%	7	N	NR	U	NR	U	0.0%						**17.5%**
Mohsen_2016	5	M	NR	U	NR	U	30.0%	5	N	10→15→20	N	NR	U	0.0%						**15.0%**
Monteiro_2026	3	M	75–90% HRmax	M	NR	U	65.0%	3	M	8	M	2	M	100.0%						**82.5%**
Ossowski_2016	3	M	50–70% HRR	M	40	M	100.0%													**100.0%**

Otero_2017	3	M	2–60 s	N	20	M	65.0 %	3	M	1–10	N	1–4	N	30.0 %						**47.5%**
Posch_2019	2	N	NR	U	45–60	M	35.0 %	2	M	NR	U	NR	U	30.0 %						**32.5%**
Riaz_2024	3	M	NR	U	30	M	65.0 %													**65.0%**
Stanghelle_2020								2	M	8–12	M	2	M	100.0 %						**100.0%**
Teixeira_2010								2	M	NR	U	NR	U	30.0 %						**30.0%**
Wen_2017	3	M	75–85 % HRR	M	40–45	M	100.0 %													**100.0%**

Note. M indicates that sufficient prescription information was reported and the reported value met the corresponding ACSM criterion; U indicates that the relevant prescription information was unclear or not reported and consistency could not be determined; N indicates that sufficient information was reported but the reported value did not meet the corresponding ACSM criterion. In the primary analysis, M receives the full component weight, whereas U and N receive zero. Total reported consistency is calculated as the mean of the included exercise-domain scores; ≥65% denotes high reported consistency and <65% denotes low or uncertain reported consistency. Warm-up or cool-down stretching is not counted as an independent flexibility prescription. Sensitivity-analysis scoring is reported separately. NR, not reported.

4. Sensitivity analyses

Sensitivity analyses examined whether the pooled effects and subgroup inferences depended on classification assumptions, individual study arms, or risk of bias. Unless otherwise specified, the outcome data, mean-difference effect measure, random-effects framework, restricted maximum likelihood estimator for between-study variance, and Hartung-Knapp confidence intervals were the same as in the primary analysis. Formal tests for subgroup differences were used to assess effect modification.

4.1 Alternative classification threshold

The classification threshold was increased from 65% to 70% while all component scores and primary scoring rules remained unchanged. Study arms were reclassified using the alternative threshold, and the subgroup meta-analyses were repeated for each outcome. This analysis assessed whether the subgroup conclusions depended on the researcher-defined 65% cut-off.

Table S5. Sensitivity analysis using a ≥70% threshold for reported consistency with ACSM recommendations

Alternative cut-off sensitivity analysis (≥70%)					
**Study**	**Primary total**	**Primary group (≥65%)**	**Reclassified group (≥70%)**	**Group change**	**Outcome**
Alkan_2011a	0	Low/uncertain	Low/uncertain	No change	TUG,BBS
Alkan_2011b	65%	High	Low/uncertain	High → Low/uncertain	TUG,BBS
Alkan_2011c	35%	Low/uncertain	Low/uncertain	No change	TUG,BBS
Çergel_2019a	70%	High	High	No change	TUG
Çergel_2019b	70%	High	High	No change	TUG
Evstigneeva_2016	30%	Low/uncertain	Low/uncertain	No change	TUG
Kanemaru_2010	0	Low/uncertain	Low/uncertain	No change	TUG
Kotsili_2025	82.5%	High	High	No change	TUG,BBS,30STS
Lee_2021	70%	High	High	No change	TUG,30STS
Madureira_2007	65%	High	Low/uncertain	High → Low/uncertain	BBS,30STS
Mewara_2024	0	Low/uncertain	Low/uncertain	No change	TUG,30STS

Miko_2018	17.5%	Low/uncertain	Low/uncertain	No change	TUG,BBS
Mohsen_2016	15%	Low/uncertain	Low/uncertain	No change	BBS
Monteiro_2026	82.5%	High	High	No change	TUG,30STS
Ossowski_2016	100%	High	High	No change	TUG
Otero_2017	47.5%	Low/uncertain	Low/uncertain	No change	TUG
Posch_2019	32.5%	Low/uncertain	Low/uncertain	No change	TUG,30STS
Riaz_2024	65%	High	Low/uncertain	High → Low/uncertain	TUG
Stanghelle_2020	100%	High	High	No change	TUG
Teixeira_2010	30%	Low/uncertain	Low/uncertain	No change	TUG,BBS
Wen_2017	100%	High	High	No change	30STS

Note. Primary total and primary group are based on the original scoring rules and the 65% threshold. Alternative group uses a threshold of at least 70% for high reported consistency. Component weights and study-arm scores were not changed. Group change indicates whether a study arm moved between classification groups.

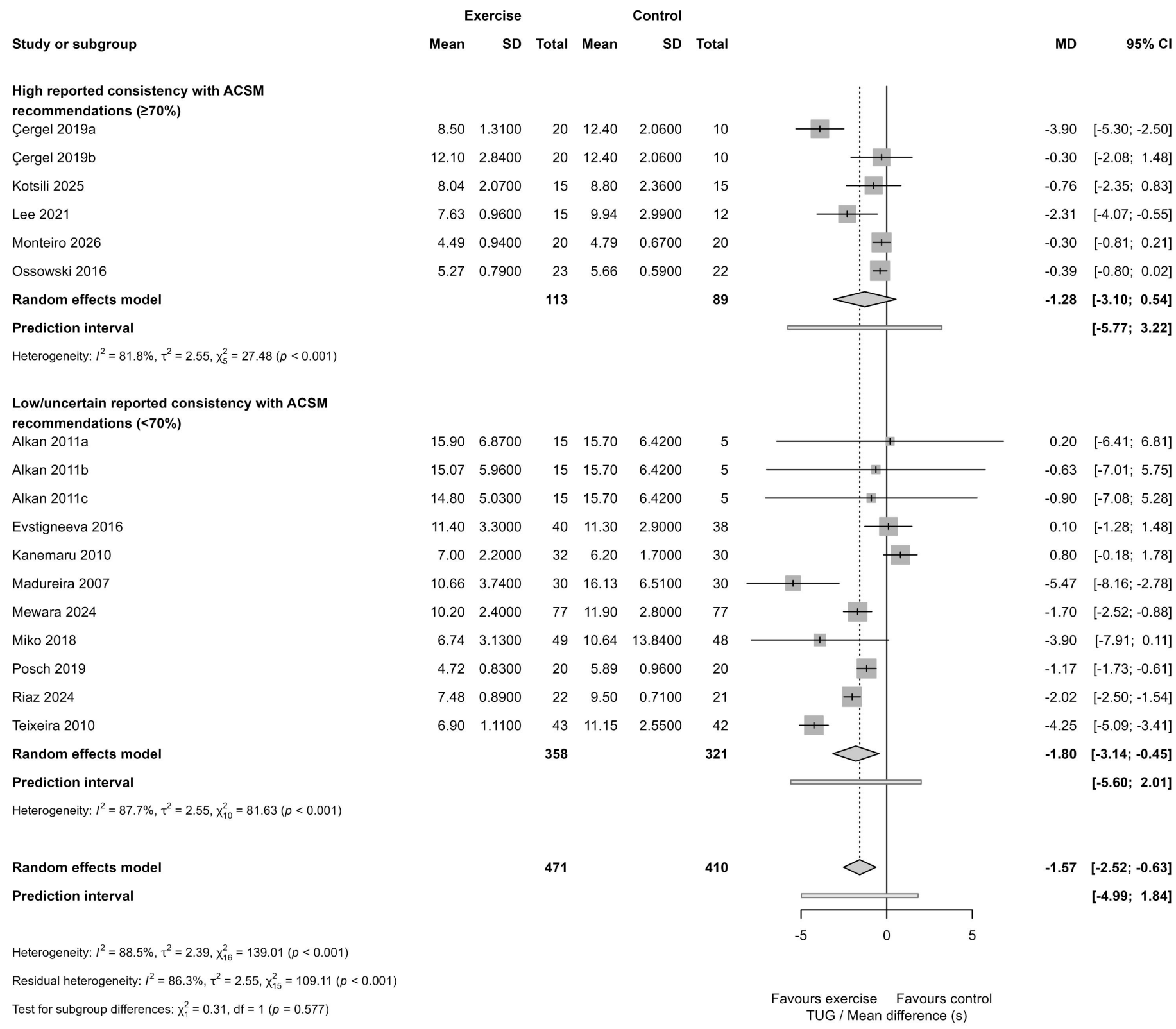

Figure S1. TUG subgroup analysis using the alternative reported-consistency threshold of ≥70%

Note. Only the classification threshold was changed. Scores of at least 70% denoted high reported consistency. Random-effects mean differences and 95% confidence intervals were calculated using REML and Hartung-Knapp inference. The subgroup-difference test evaluates the interaction between reported-consistency groups. Negative mean differences favour exercise for TUG.

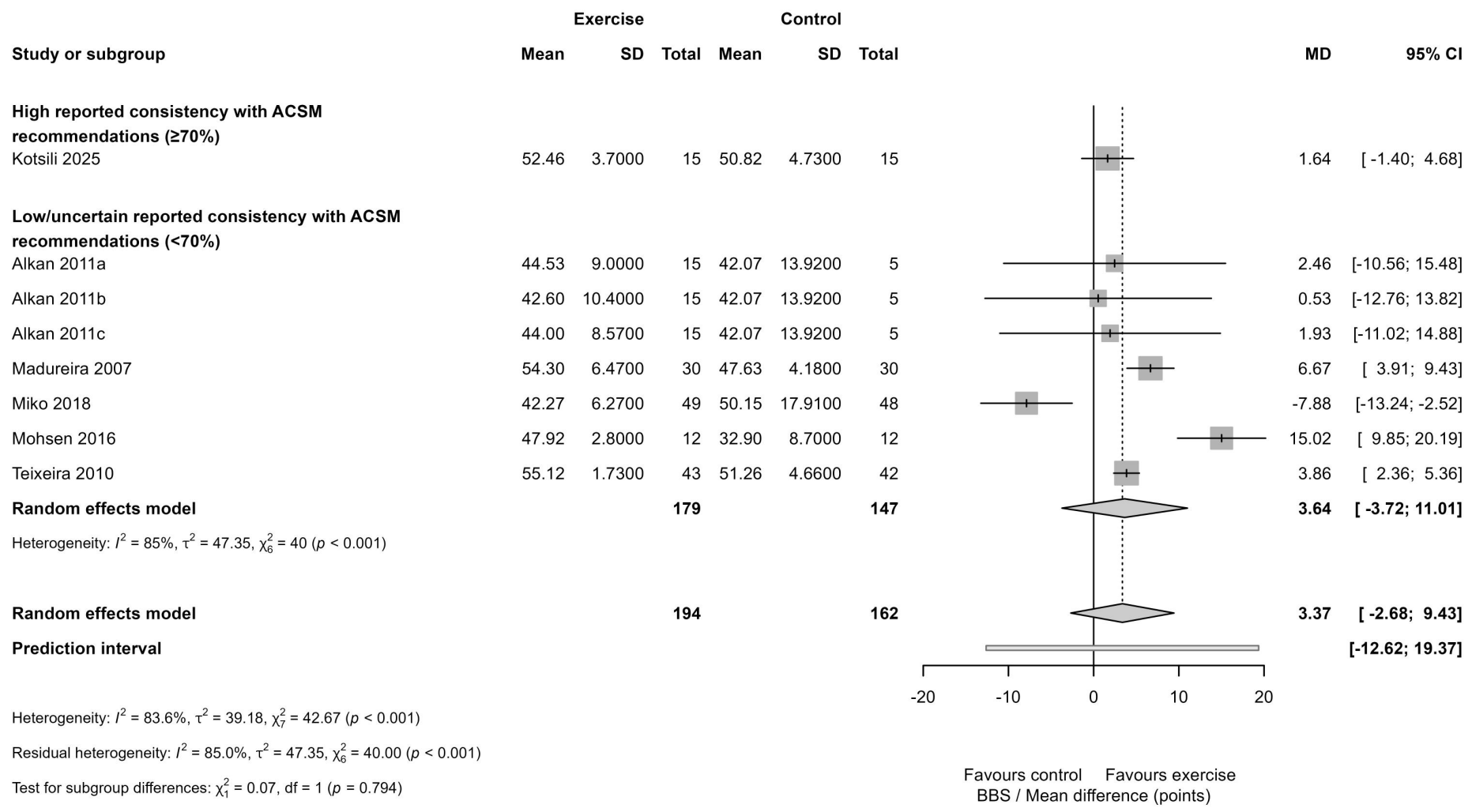

Figure S2. BBS subgroup analysis using the alternative reported-consistency threshold of ≥70%

Note. Only the classification threshold was changed. Scores of at least 70% denoted high reported consistency. Random-effects mean differences and 95% confidence intervals were calculated using REML and Hartung-Knapp inference. The subgroup-difference test evaluates the interaction between reported-consistency groups. Positive mean differences favour exercise for BBS.

4.2 Alternative half-weight scoring of unclear or unreported exercise-prescription components

To distinguish insufficient reporting of exercise-prescription components from clear non-fulfilment of the ACSM prescription criteria, components classified as U because the relevant information was unclear or not reported were assigned half of their corresponding prespecified weight. Components classified as M retained the full weight, whereas components classified as N received zero. The original threshold of ≥65% for high reported consistency with ACSM recommendations was retained.

Study-arm exercise-prescription scores and reported-consistency classifications were recalculated before repeating the subgroup meta-analyses. Several TUG and BBS study arms changed classification groups, whereas no 30STS study arm was reclassified. Therefore, the 30STS subgroup result was identical to the primary analysis and was not duplicated in a separate forest plot.

Table S6. Sensitivity analysis using half-weight scoring for unclear or unreported exercise-prescription components

Sensitivity analysis of reported consistency with ACSM recommendations using alternative half-weight scoring								
**Study arm**	**Primary total**	**Primary group**	**Balance-oriented weight-bearing/aerobic activity**	**Resistance sensitivity score**	**Flexibility sensitivity score**	**Sensitivity total**	**Sensitivity group**	**Group change**
Alkan_2011a	0	Low/uncertain	35			35%	Low/uncertain	No change
Alkan_2011b	65%	High	82.5%			82.5%	High	No change
Alkan_2011c	35%	Low/uncertain	52.5%			52.5%	Low/uncertain	No change
Çergel_2019a	70%	High		70%		70%	High	No change
Çergel_2019b	70%	High		70%		70%	High	No change
Evstigneeva_2016	30%	Low/uncertain		65%		65%	High	Low/uncertain → High

Sensitivity analysis of reported consistency with ACSM recommendations using alternative half-weight scoring								
**Study arm**	**Primary total**	**Primary group**	**Balance-oriented weight-bearing/aerobic activity**	**Resistance sensitivity score**	**Flexibility sensitivity score**	**Sensitivity total**	**Sensitivity group**	**Group change**
Kanemaru_2010	0	Low/uncertain		35%		35%	Low/uncertain	No change
Kotsili_2025	82.5%	High	65%	100%		82.5%	High	No change
Lee_2021	70%	High		70%		70%	High	No change
Madureira_2007	65%	High	65%			65%	High	No change
Mewara_2024	0	Low/uncertain	50%	50%	0.5	50%	Low/uncertain	No change
Miko_2018	17.5%	Low/uncertain	52.5%	35%		43.75%	Low/uncertain	No change
Mohsen_2016	15%	Low/uncertain	65%	15%		40%	Low/uncertain	No change
Monteiro_2026	82.5%	High	82.5%	100%		91.25%	High	No change
Ossowski_2016	100%	High	100%			100%	High	No change
Otero_2017	47.5%	Low/uncertain	65%	30%		47.5%	Low/uncertain	No change
Posch_2019	32.5%	Low/uncertain	52.5%	65%		58.75%	Low/uncertain	No change
Riaz_2024	65%	High	82.5%			82.5%	High	No change
Stanghelle_2020	100%	High		100%		100%	High	No change
Teixeira_2010	30%	Low/uncertain		65%		65%	High	Low/uncertain → High
Wen_2017	100%	High	100%			100%	High	No change

Note. Primary total and primary group were calculated using the primary scoring rule, in which components classified as M received the full prespecified weight, whereas components classified as U or N received zero. In the alternative scoring analysis, components classified as U received half of their corresponding prespecified weight, components classified as M retained the full weight, and components classified as N remained assigned zero. The threshold of ≥65% for high reported consistency with ACSM recommendations was unchanged. Group change identifies study arms that were reclassified under the alternative scoring method.

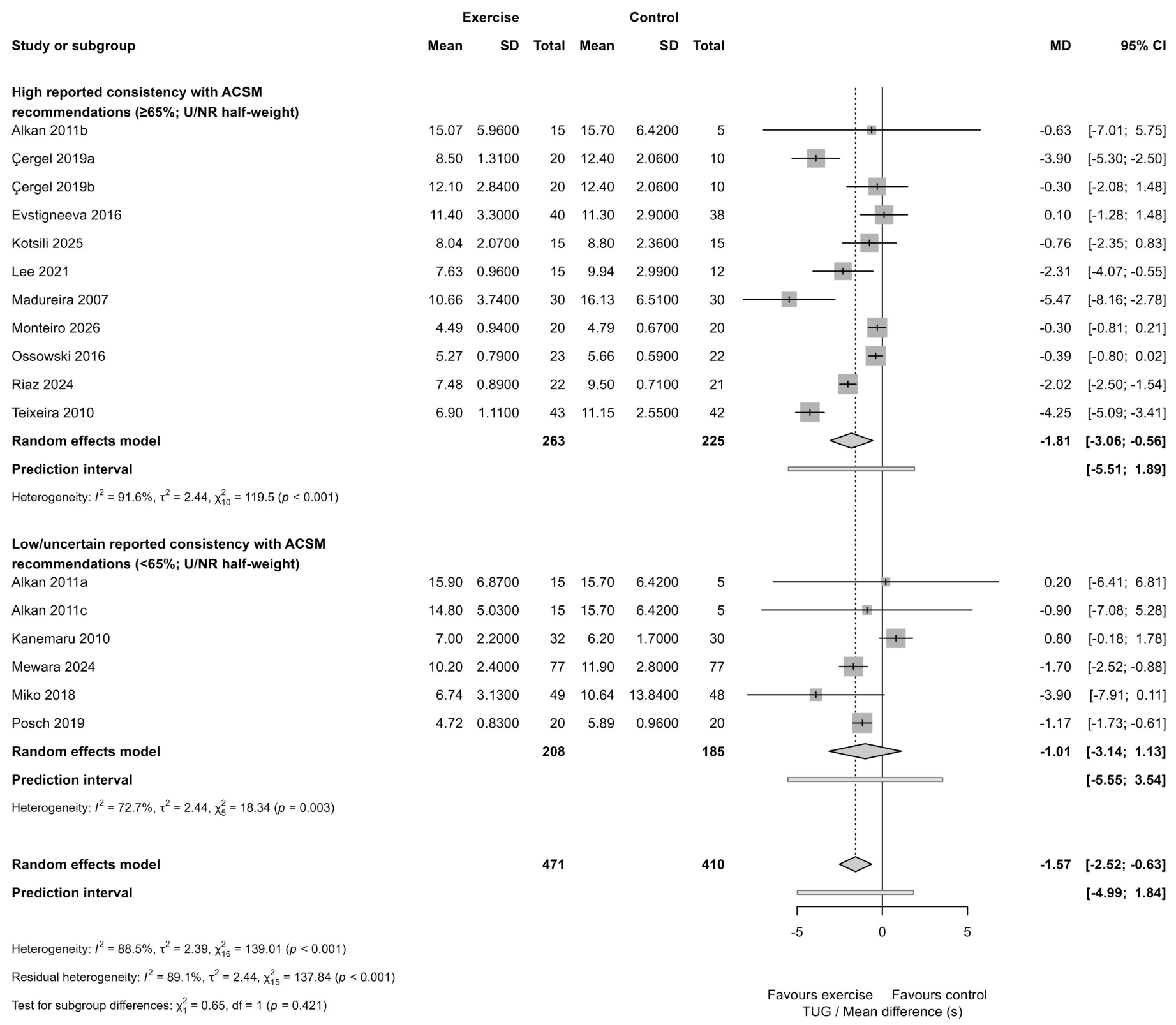

Figure S3. TUG subgroup analysis using half-weight scoring for unclear or unreported exercise-prescription components

Note. Exercise-prescription components classified as U because the relevant information was unclear or not reported received half of their corresponding prespecified weight. Components classified as M retained the full weight, whereas components classified as N received zero. The threshold of ≥65% for high reported consistency with ACSM recommendations was retained. Random-effects mean differences and 95% confidence intervals were calculated using REML and Hartung–Knapp inference. The subgroup-difference test evaluates the interaction between the reported-consistency groups. Negative mean differences favour exercise for TUG.

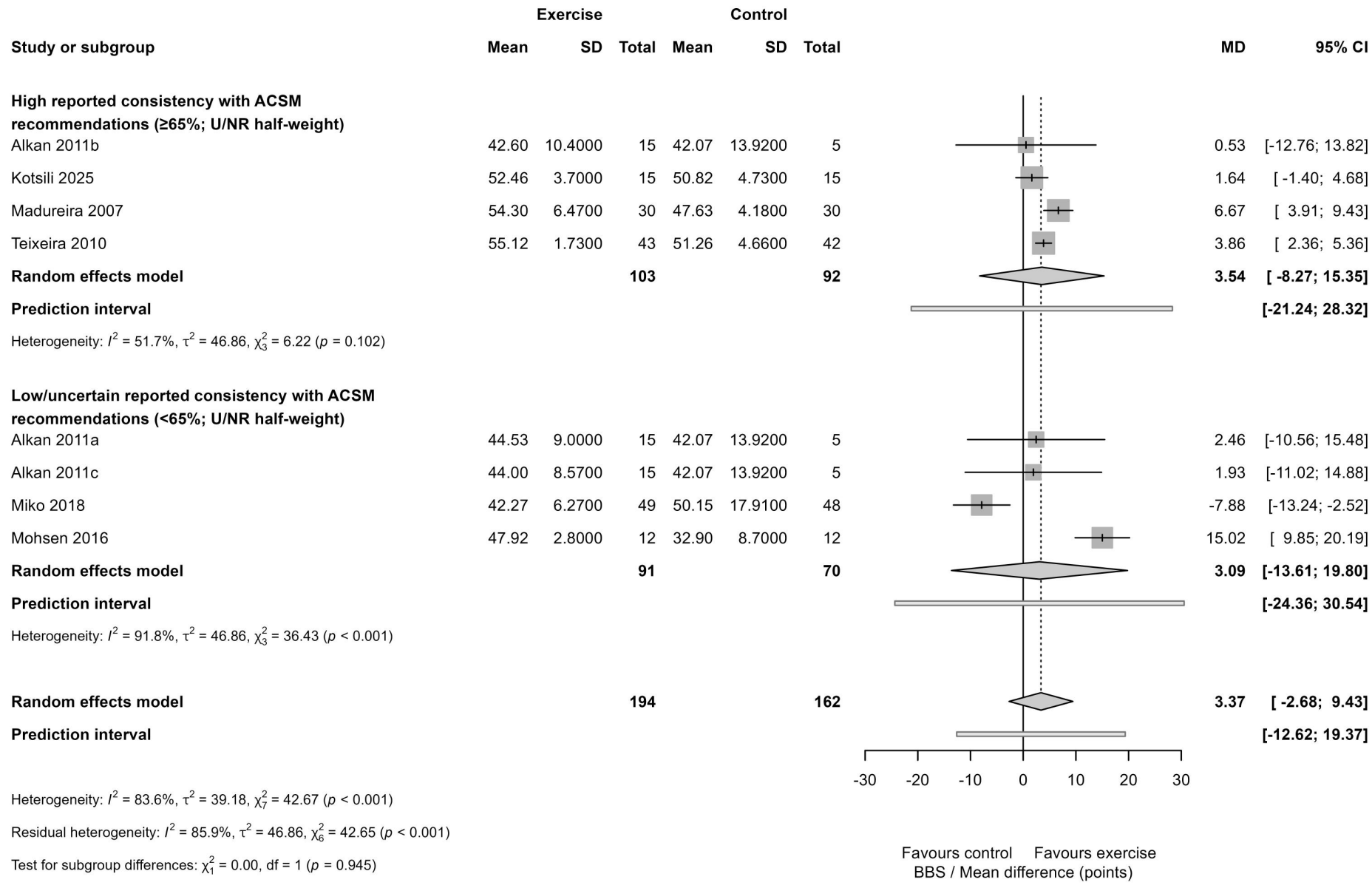

Figure S4. BBS subgroup analysis using half-weight scoring for unclear or unreported exercise-prescription components

Note. Exercise-prescription components classified as U because the relevant information was unclear or not reported received half of their corresponding prespecified weight. Components classified as M retained the full weight, whereas components classified as N received zero. The threshold of ≥65% for high reported consistency with ACSM recommendations was retained. Random-effects mean differences and 95% confidence intervals were calculated using REML and Hartung–Knapp inference. The subgroup-difference test evaluates the interaction between the reported-consistency groups. Positive mean differences favour exercise for BBS.

4.3 Leave-one-study-arm-out analyses

Each eligible study arm was removed in turn, and the overall random-effects meta-analysis was refitted using the remaining comparisons. Changes in the pooled estimate, confidence interval, between-study variance, and heterogeneity were examined to identify influential study arms. The row labelled “All study arms” represents the analysis before omission.

4.3.1 TUG

Table S7. Leave-one-study-arm-out sensitivity analysis for TUG

study	MD (95% CI)	P value	Tau2	I2 (%)	Q statistic	P for heterogeneity
Alkan 2011a	-1.60 [-2.56, -0.64]	0.003	2.4347	89.2	138.85	<0.001
Alkan 2011b	-1.59 [-2.56, -0.63]	0.003	2.443	89.2	138.98	<0.001
Alkan 2011c	-1.59 [-2.55, -0.62]	0.003	2.4468	89.2	139	<0.001
Çergel 2019a	-1.40 [-2.33, -0.46]	0.006	2.1188	87.9	124.09	<0.001
Çergel 2019b	-1.66 [-2.66, -0.66]	0.003	2.5052	89.1	138.08	<0.001
Evstigneeva 2016	-1.70 [-2.68, -0.71]	0.002	2.4034	88.9	135.67	<0.001
Kanemaru 2010	-1.76 [-2.69, -0.83]	0.001	2.077	87.8	122.6	<0.001
Kotsili 2025	-1.64 [-2.65, -0.62]	0.004	2.5733	89.2	138.75	<0.001
Lee 2021	-1.53 [-2.54, -0.52]	0.006	2.5593	89.1	137.38	<0.001
Madureira 2007	-1.38 [-2.28, -0.48]	0.005	1.9947	88.4	129.11	<0.001
Mewara 2024	-1.57 [-2.60, -0.54]	0.005	2.6514	89.1	137.32	<0.001
Miko 2018	-1.50 [-2.47, -0.54]	0.005	2.4003	89.1	137.23	<0.001
Monteiro 2026	-1.69 [-2.69, -0.68]	0.003	2.4845	88	125.41	<0.001
Ossowski 2016	-1.68 [-2.69, -0.67]	0.003	2.5097	87.5	120	<0.001
Posch 2019	-1.62 [-2.65, -0.58]	0.004	2.6497	89.2	139.01	<0.001
Riaz 2024	-1.54 [-2.57, -0.51]	0.006	2.6357	87.9	124.3	<0.001
Teixeira 2010	-1.32 [-2.18, -0.45]	0.005	1.723	82.2	84.05	<0.001
All study arms included	-1.57 [-2.52, -0.63]	0.003	2.3908	88.5	139.01	<0.001

Note. TUG, Timed Up and Go test; MD, mean difference; CI, confidence interval. Negative MD values favour exercise because a shorter TUG time indicates better performance. Each row shows the pooled result after omitting the corresponding study arm; the final row shows the analysis including all study arms. Tau^2 was estimated using REML, and 95% CIs were calculated using the Hartung-Knapp method.

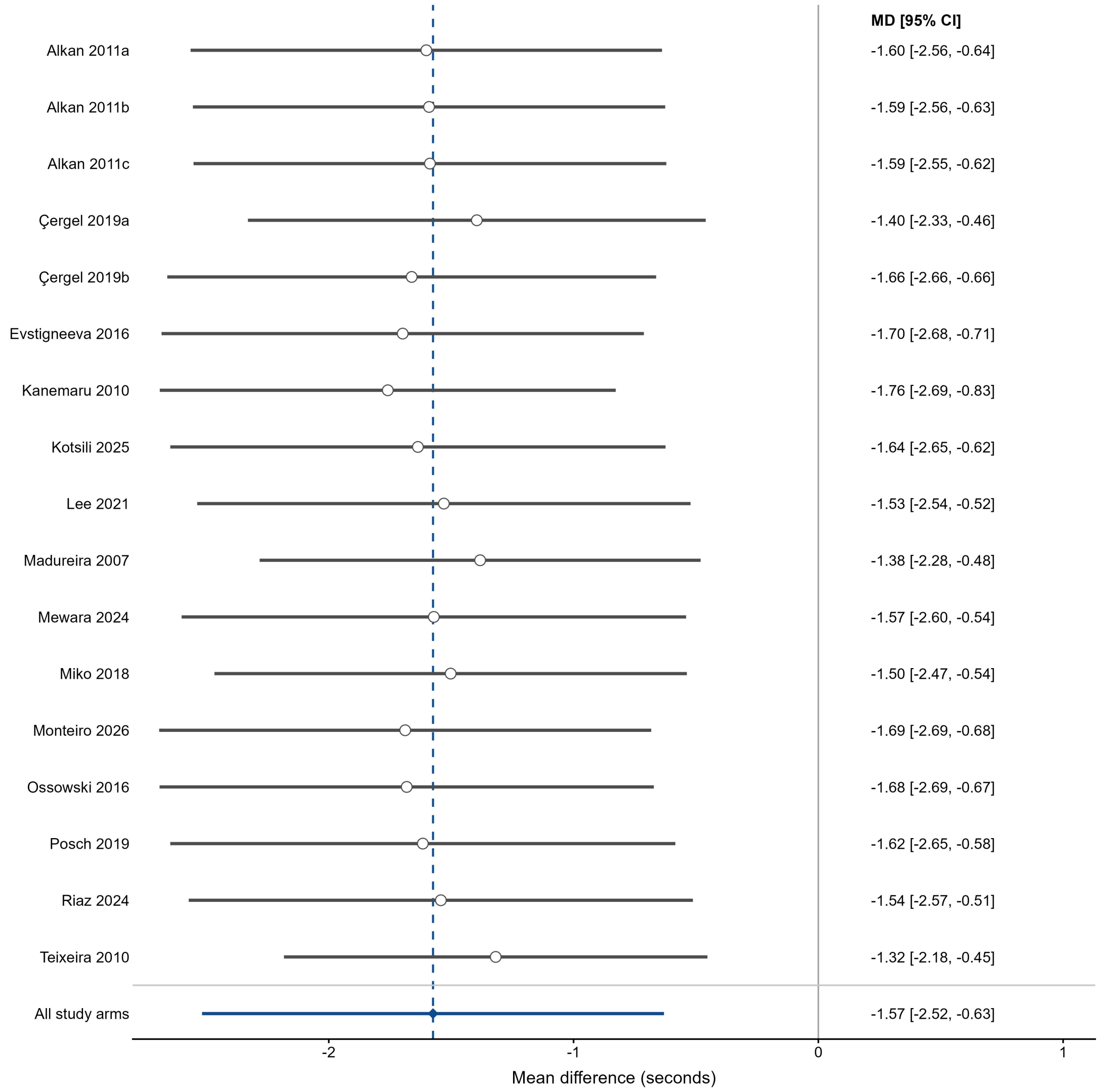

Figure S5. Leave-one-study-arm-out sensitivity analysis for TUG

Note. Each point shows the pooled mean difference after omission of the named study arm; the estimate for all study arms is shown for reference. Horizontal lines represent 95% confidence intervals. Negative mean differences favour exercise for TUG. REML, restricted maximum likelihood.

4.3.2 BBS

Table S8. Leave-one-study-arm-out sensitivity analysis for BBS

study	MD (95% CI)	P value	Tau2	I2 (%)	Q statistic	P for heterogeneity
Alkan 2011a	3.43 [-3.42, 10.28]	0.267	43.9353	85.9	42.62	<0.001
Alkan 2011b	3.60 [-3.21, 10.41]	0.243	43.3231	85.9	42.41	<0.001
Alkan 2011c	3.48 [-3.37, 10.33]	0.26	43.8599	85.9	42.57	<0.001
Kotsili 2025	3.64 [-3.72, 11.01]	0.271	47.3521	85	40	<0.001
Madureira 2007	2.73 [-4.54, 10.00]	0.394	45.7471	84.3	38.29	<0.001

Miko 2018	5.40 [0.34, 10.46]	0.04	18.723	73.9	22.96	<0.001
Mohsen 2016	1.60 [-3.62, 6.81]	0.482	20.1768	75.3	24.3	<0.001
Teixeira 2010	3.23 [-4.23, 10.70]	0.33	48.5776	85.9	42.6	<0.001
All study arms included	3.37 [-2.68, 9.43]	0.229	39.1849	83.6	42.67	<0.001

Note. BBS, Berg Balance Scale; MD, mean difference; CI, confidence interval. Positive MD values favour exercise because a higher BBS score indicates better balance performance. Each row shows the pooled result after omitting the corresponding study arm; the final row shows the analysis including all study arms. Between-study variance (τ^2) was estimated using REML, and 95% CIs were calculated using Hartung-Knapp inference.

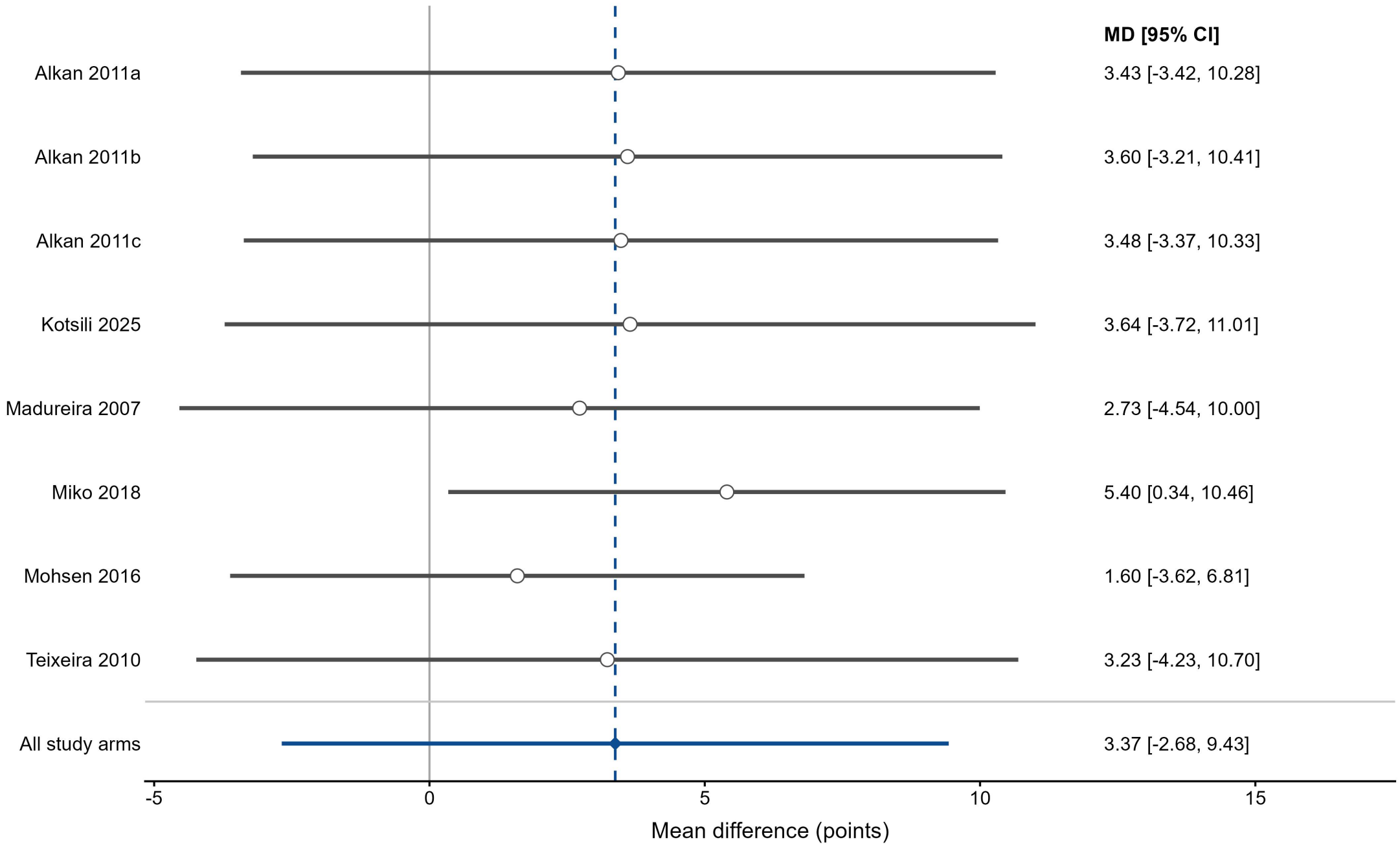

Figure S6. Leave-one-study-arm-out sensitivity analysis for BBS

Note. Each point shows the pooled mean difference after omission of the named study arm; the estimate for all study arms is shown for reference. Horizontal lines represent 95% confidence intervals. Positive mean differences favour exercise for BBS. REML, restricted maximum likelihood.

4.3.3 30STS

Table S9. Leave-one-study-arm-out sensitivity analysis for 30STS

study	MD (95% CI)	P value	Tau2	I2 (%)	Q statistic	P for heterogeneity
Kotsili 2025	4.16 [1.94, 6.39]	0.004	4.3544	88.7	53.09	<0.001
Lee 2021	3.89 [1.65, 6.13]	0.005	4.6985	89.4	56.36	<0.001
Mewara 2024	4.10 [1.80, 6.40]	0.005	4.6592	88.5	52.09	<0.001
Monteiro 2026	3.88 [1.68, 6.07]	0.005	4.5785	89.4	56.35	<0.001
Otero 2017	2.87 [1.70, 4.04]	<0.001	0.5639	31.5	8.76	0.188

Posch 2019	3.79 [1.49, 6.08]	0.007	4.7817	89.2	55.56	<0.001
Stanghelle 2020	4.29 [2.16, 6.41]	0.003	3.8194	86	42.73	<0.001
Wen 2017	3.81 [1.57, 6.06]	0.006	4.68	89.3	56.06	<0.001
All study arms included	3.88 [1.93, 5.83]	0.002	4.1347	87.6	56.37	<0.001

Note. 30STS, 30-second sit-to-stand test; MD, mean difference; CI, confidence interval. Positive MD values favour exercise because a higher 30STS score indicates better lower-limb function. Each row shows the pooled result after omitting the corresponding study arm; the final row shows the analysis including all study arms. Between-study variance (τ^2) was estimated using REML, and 95% CIs were calculated using Hartung-Knapp inference.

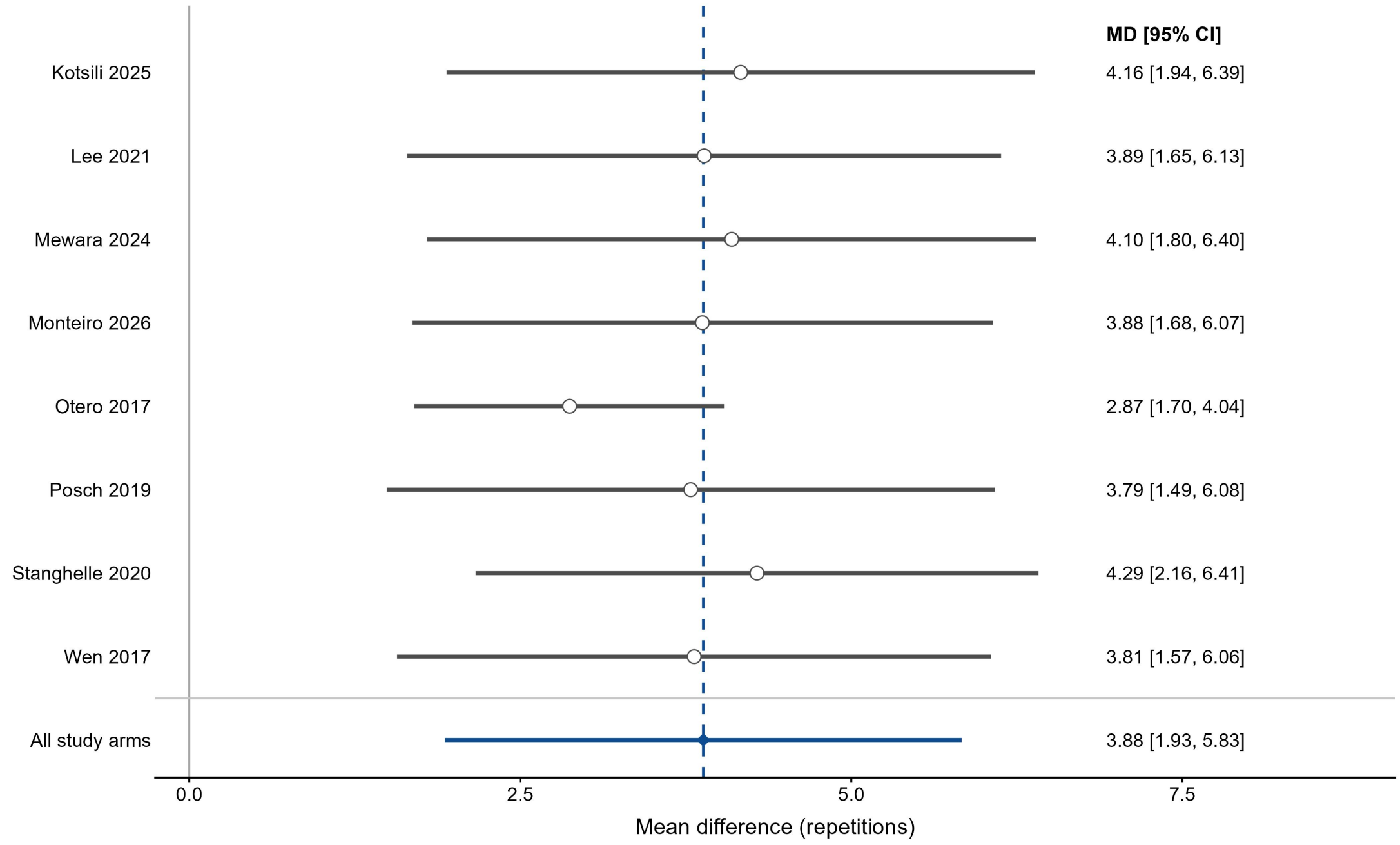

Figure S7. Leave-one-study-arm-out sensitivity analysis for 30STS

Note. Each point shows the pooled mean difference after omission of the named study arm; the estimate for all study arms is shown for reference. Horizontal lines represent 95% confidence intervals. Positive mean differences favour exercise for 30STS. REML, restricted maximum likelihood.

4.4 Exclusion of studies at high risk of bias

For outcomes with eligible studies judged to be at high risk of bias under the outcome-specific RoB 2 assessment, the meta-analysis was repeated after those studies were removed. All other statistical settings and the 65% reported-consistency threshold were retained.

4.4.1 TUG

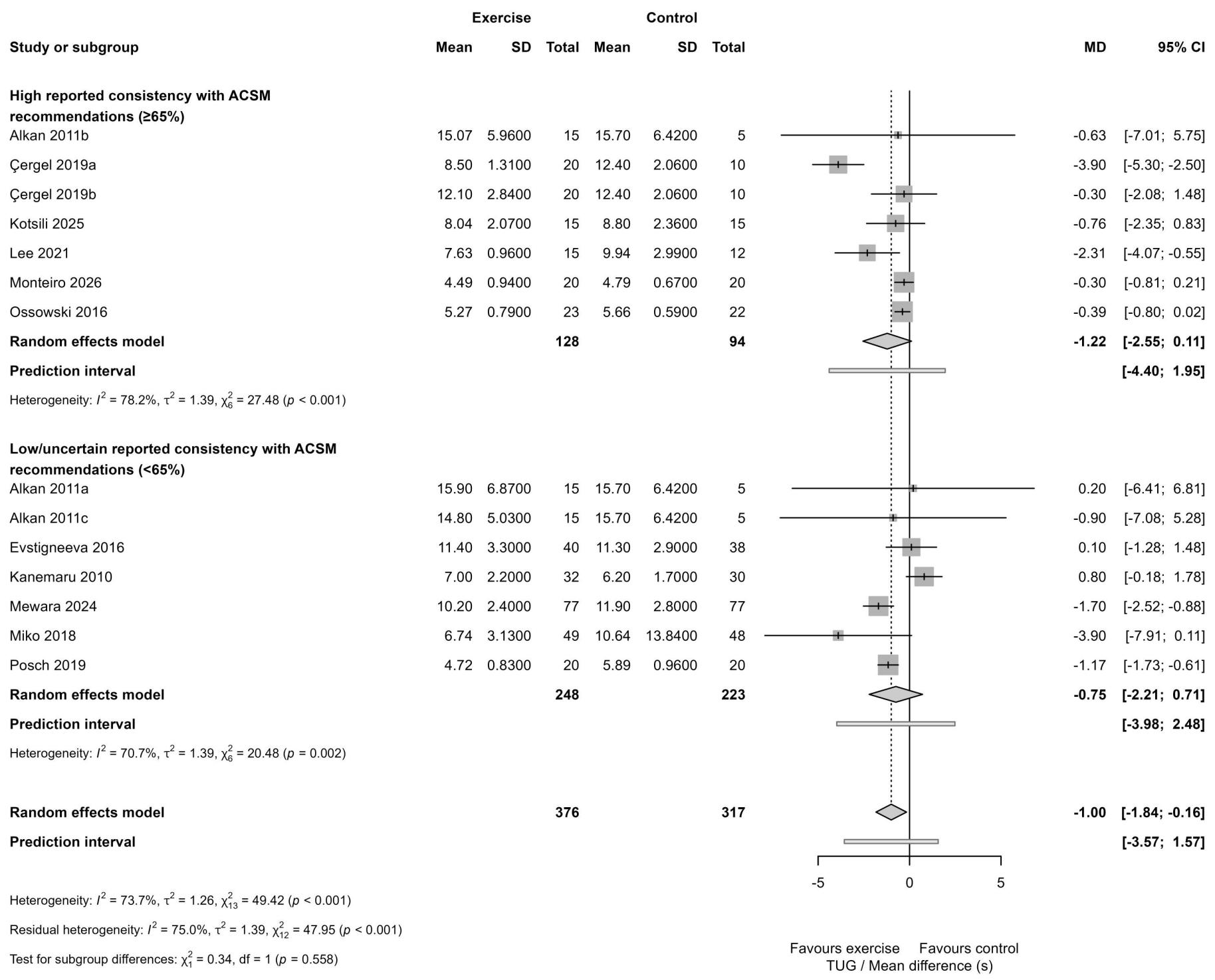

Figure S8. TUG sensitivity analysis after excluding studies at high risk of bias

Note. Studies judged to be at high risk of bias for the TUG outcome were excluded. Random-effects mean differences and 95% confidence intervals were calculated using REML and Hartung-Knapp inference. Negative mean differences favour exercise. RoB 2, revised Cochrane risk-of-bias tool for randomised trials.

4.4.2 BBS

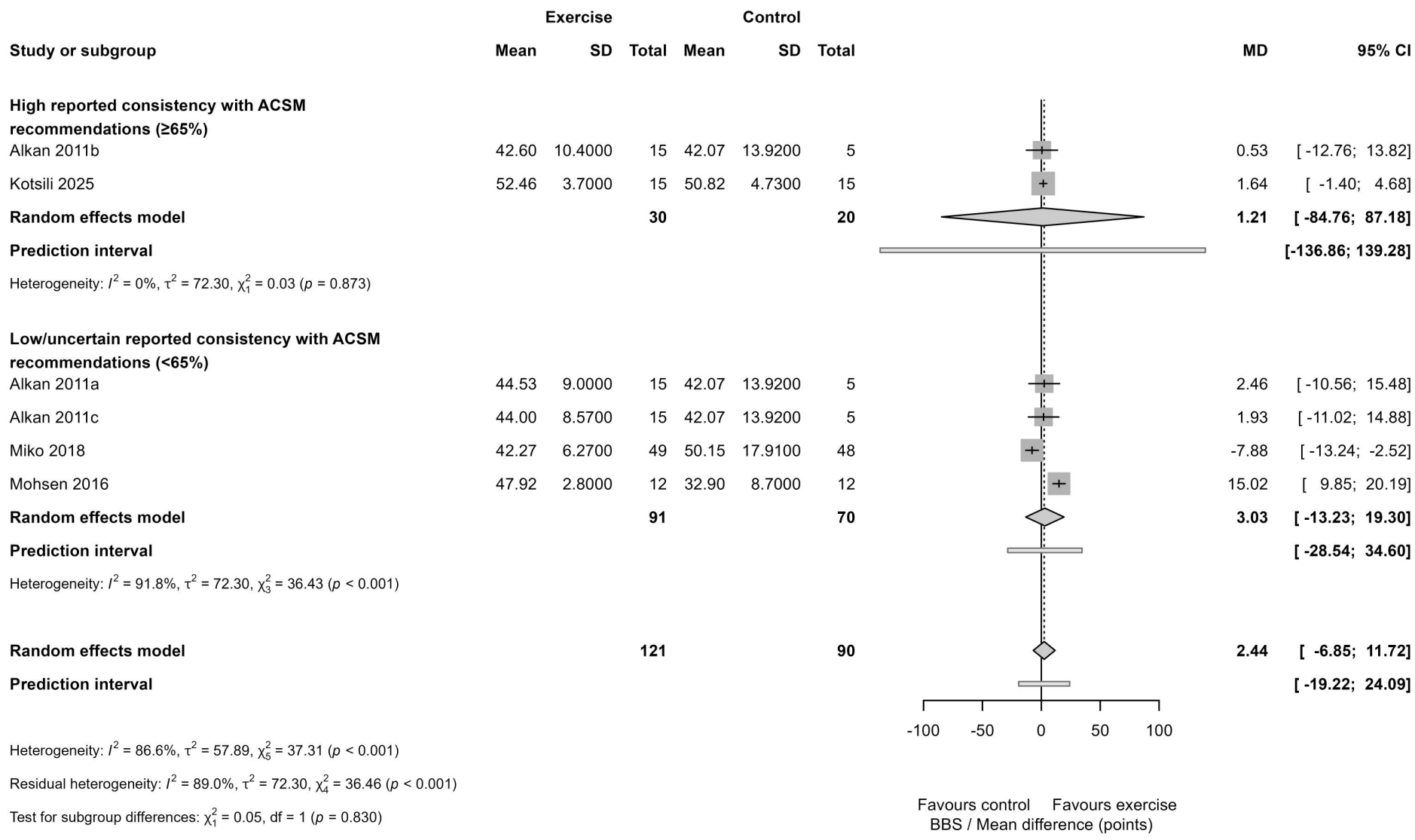

Figure S9. BBS sensitivity analysis after excluding studies at high risk of bias

Note. Studies judged to be at high risk of bias for the BBS outcome were excluded. Random-effects mean differences and 95% confidence intervals were calculated using REML and Hartung-Knapp inference. Positive mean differences favour exercise. RoB 2, revised Cochrane risk-of-bias tool for randomised trials.

5. Small-study effects and funnel-plot asymmetry

Potential small-study effects were explored by visual inspection of funnel plots together with Egger's regression test and the Begg-Mazumdar rank-correlation test. The tests were applied to the overall outcome meta-analysis, while plotting symbols distinguish the reported-consistency classification of each study arm. Because BBS and 30STS included fewer than ten effect estimates, their asymmetry tests were considered underpowered and exploratory. A non-significant test was not interpreted as evidence that publication bias was absent.

5.1 TUG

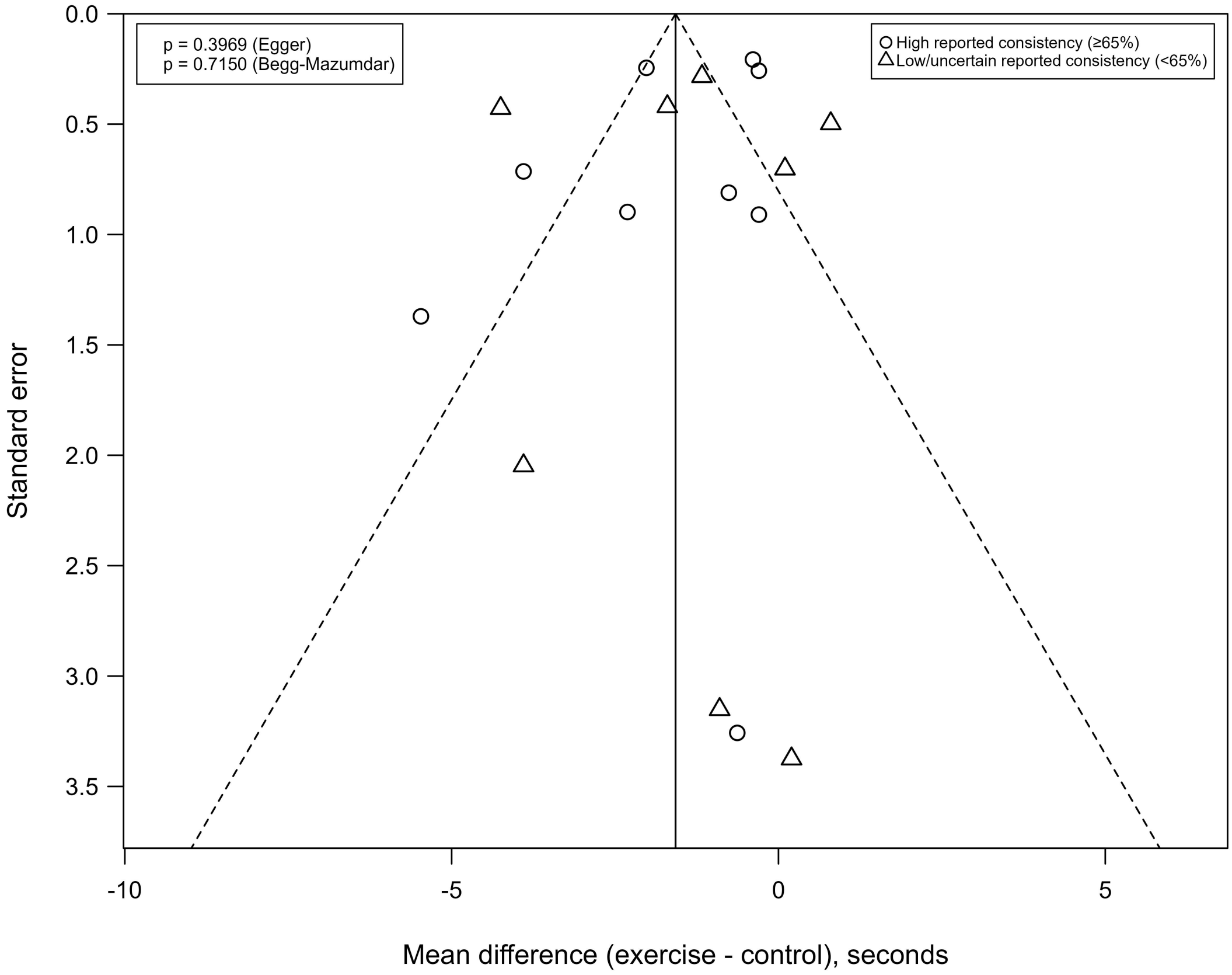

Figure S10. Funnel plot for TUG

Note. The funnel plot displays the study-arm mean difference against its standard error. The vertical line represents the pooled effect, and diagonal lines represent approximate 95% funnel limits. Circles and triangles distinguish high and low or uncertain reported consistency with ACSM recommendations. Egger and Begg-Mazumdar P values are shown in the figure. Negative mean differences favour exercise for TUG.

5.2 BBS

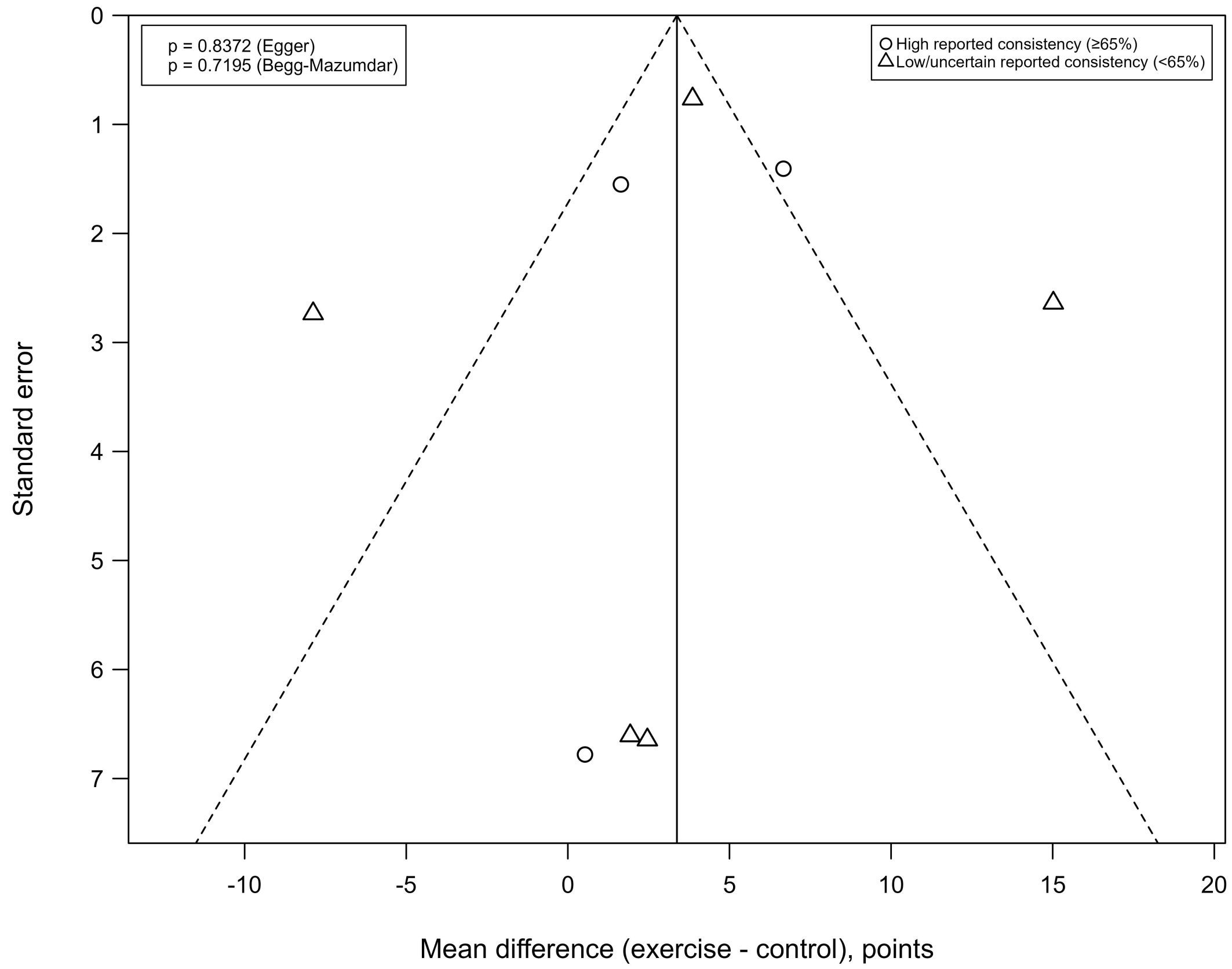

Figure S11. Funnel plot for BBS

Note. The funnel plot displays the study-arm mean difference against its standard error. The vertical line represents the pooled effect, and diagonal lines represent approximate 95% funnel limits. Circles and triangles distinguish high and low or uncertain reported consistency with ACSM recommendations. Egger and Begg-Mazumdar P values are shown in the figure. Positive mean differences favour exercise for BBS; asymmetry tests are exploratory because fewer than ten effect estimates were available.

5.3 30STS

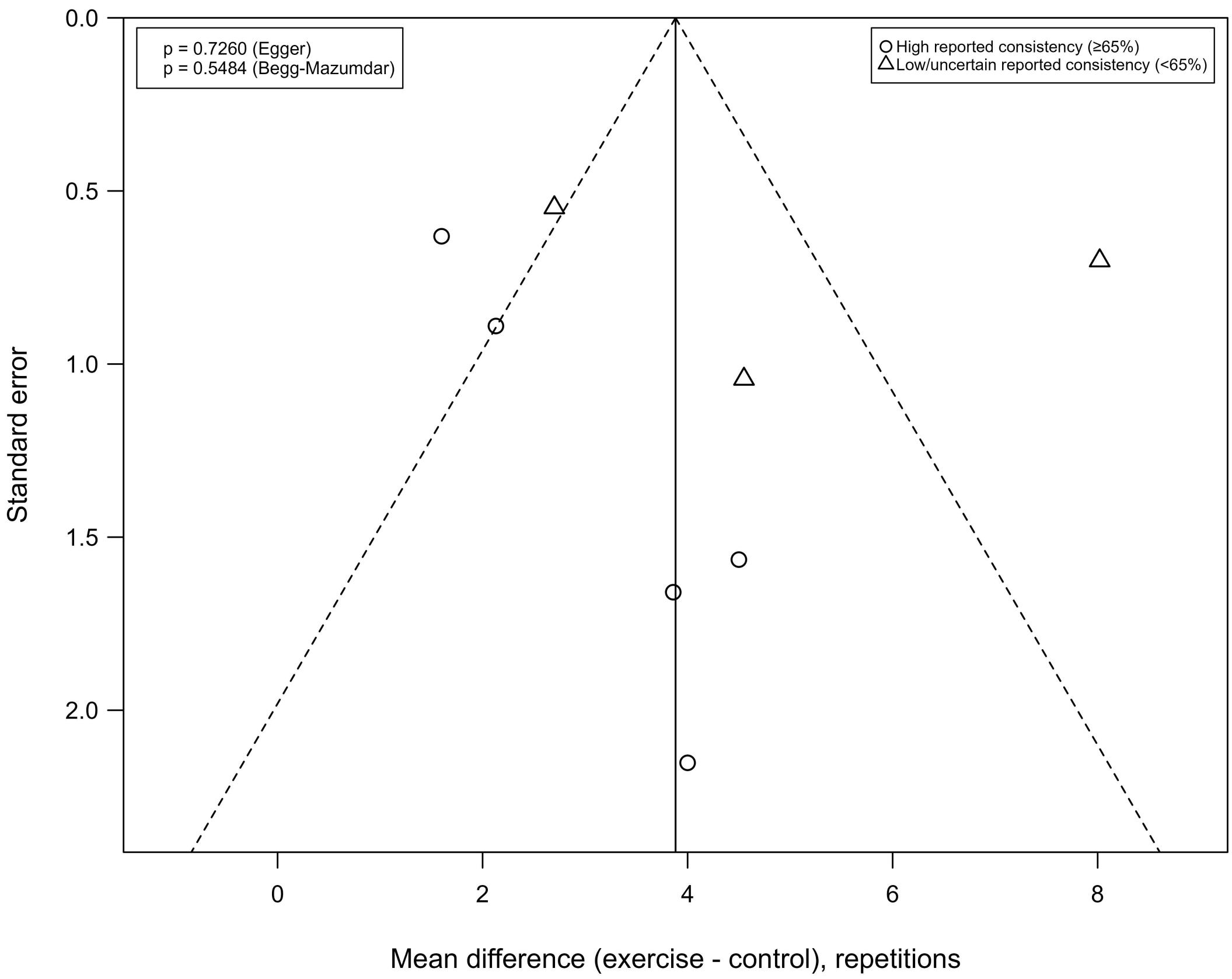

Figure S12. Funnel plot for 30STS

Note. The funnel plot displays the study-arm mean difference against its standard error. The vertical line represents the pooled effect, and diagonal lines represent approximate 95% funnel limits. Circles and triangles distinguish high and low or uncertain reported consistency with ACSM recommendations. Egger and Begg-Mazumdar P values are shown in the figure. Positive mean differences favour exercise for 30STS; asymmetry tests are exploratory because fewer than ten effect estimates were available.

6. Exploratory univariable meta-regression analyses

Potential sources of heterogeneity were examined using separate univariable random-effects meta-regression models. Each moderator was analysed independently. Study arms with missing or unreported data for a given moderator were excluded only from that model, so the number of effect estimates could differ across analyses.

Between-study variance was estimated using restricted maximum likelihood, and Hartung-Knapp inference was used for coefficient confidence intervals and P values. Results are reported as the regression coefficient, 95% confidence interval, P value, residual τ^2, and pseudo-R^2 relative to a complete-case intercept-only model. Because few effect estimates informed several models, all meta-regression analyses were treated as exploratory and were not interpreted causally.

6.1 TUG

Table S10. Univariable meta-regression analysis for TUG

Exploratory univariable meta-regression analyses for TUG						
**Moderator**	**k**	**Beta (95% CI)**	**P value**	**Pseudo-R2 (%)**	**Residual tau2**	**Residual I2 (%)**
Combined mean age	16	0.09 (-0.09 ,0.27)	0.323	5.5	2.348	89.4
Intervention duration	17	0.01 (-0.05, 0.06)	0.779	0	2.504	92
Weekly exercise frequency	16	0.38 (-0.30 , 1.05)	0.248	6.1	2.489	92.2

Note. Each moderator was examined in a separate random-effects meta-regression using REML and Hartung-Knapp inference. Beta is the change in the post-intervention mean difference (exercise minus control) per unit shown. Because negative mean differences favour exercise for TUG, a negative beta represents a shift toward greater exercise benefit as the moderator increases. Pseudo-R^2 is the proportion of between-study heterogeneity explained relative to the corresponding complete-case intercept-only model. All analyses are exploratory because of the small number of effect estimates. TUG, Timed Up and Go test; CI, confidence interval; REML, restricted maximum likelihood.

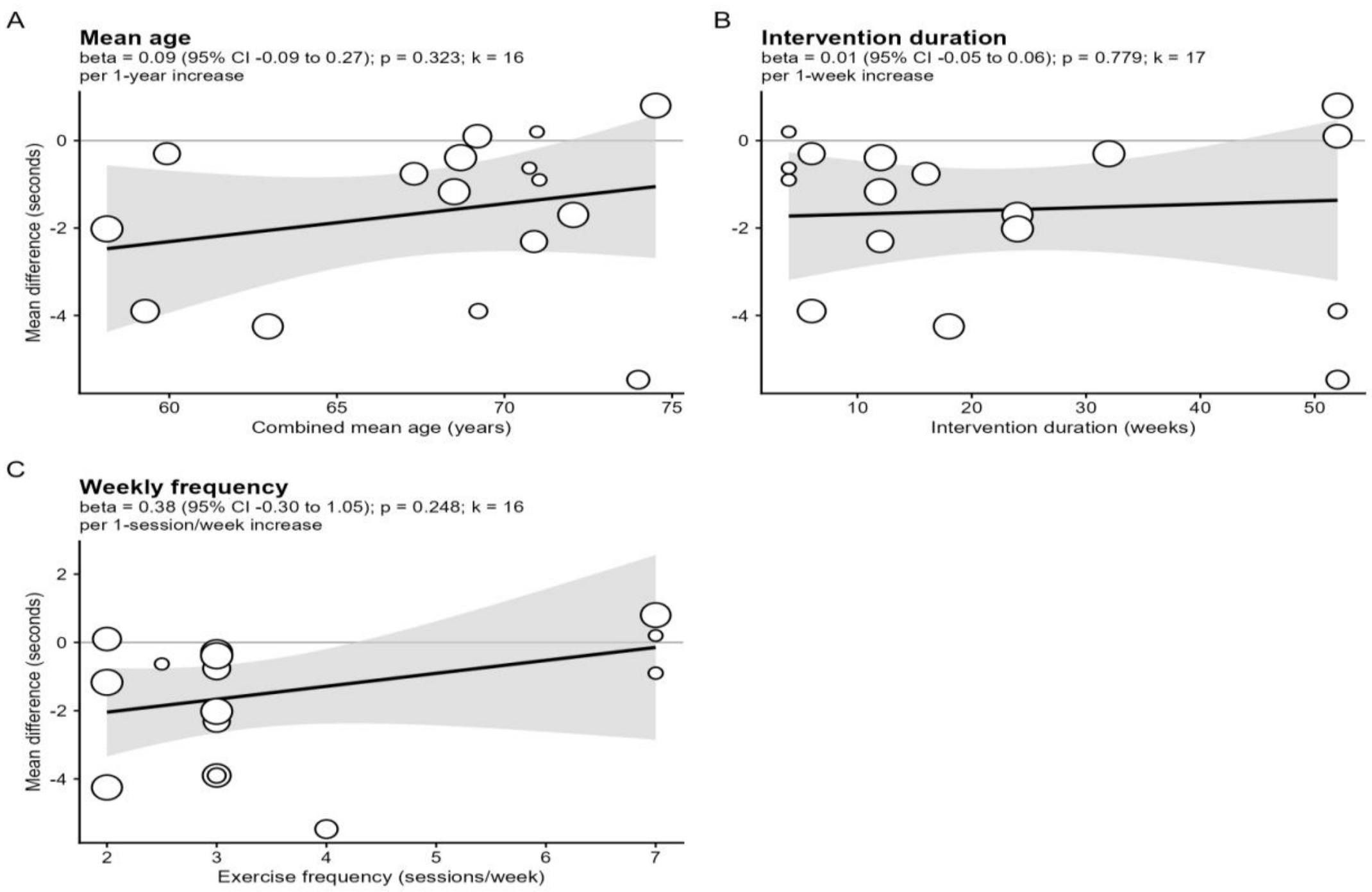

Figure S13. Univariable meta-regression analyses for TUG

Note. TUG, Timed Up and Go test; MD, mean difference; CI, confidence interval. Negative MD values favour exercise. Bubble size reflects inverse-variance study weight. Each moderator was analysed in a separate univariable random-effects meta-regression using REML and Hartung-Knapp inference. Shaded bands represent 95% confidence intervals for the fitted regression line.

6.2 BBS

Table S11. Univariable meta-regression analysis for BBS

Exploratory univariable meta-regression analyses for BBS							
**Moderator**	**Beta unit**	**k**	**Beta (95% CI)**	**P value**	**Pseudo-R2 (%)**	**Residual tau2**	**Residual I2 (%)**
Combined mean age	per 1-year increase	8	-0.64 (-1.76 ,0.49)	0.216	5.6	36.976	87.5
Intervention duration	per 1-week increase	8	-0.13 (-0.42 ,0.17)	0.331	2.2	38.306	89.4
Weekly exercise frequency	per 1-session/week increase	8	1.16 (-2.52 , 4.85)	0.470	0	39.435	88.6

Note. Each moderator was examined in a separate random-effects meta-regression using REML and Hartung-Knapp inference. Beta is the change in the post-intervention mean difference (exercise minus control) per unit shown. Positive beta values represent a shift toward greater exercise benefit for BBS. Pseudo-R^2 is the proportion of between-study heterogeneity explained relative to the corresponding complete-case intercept-only model. All analyses are exploratory because of the small number of effect estimates. BBS, Berg Balance Scale; CI, confidence interval; REML, restricted maximum likelihood.

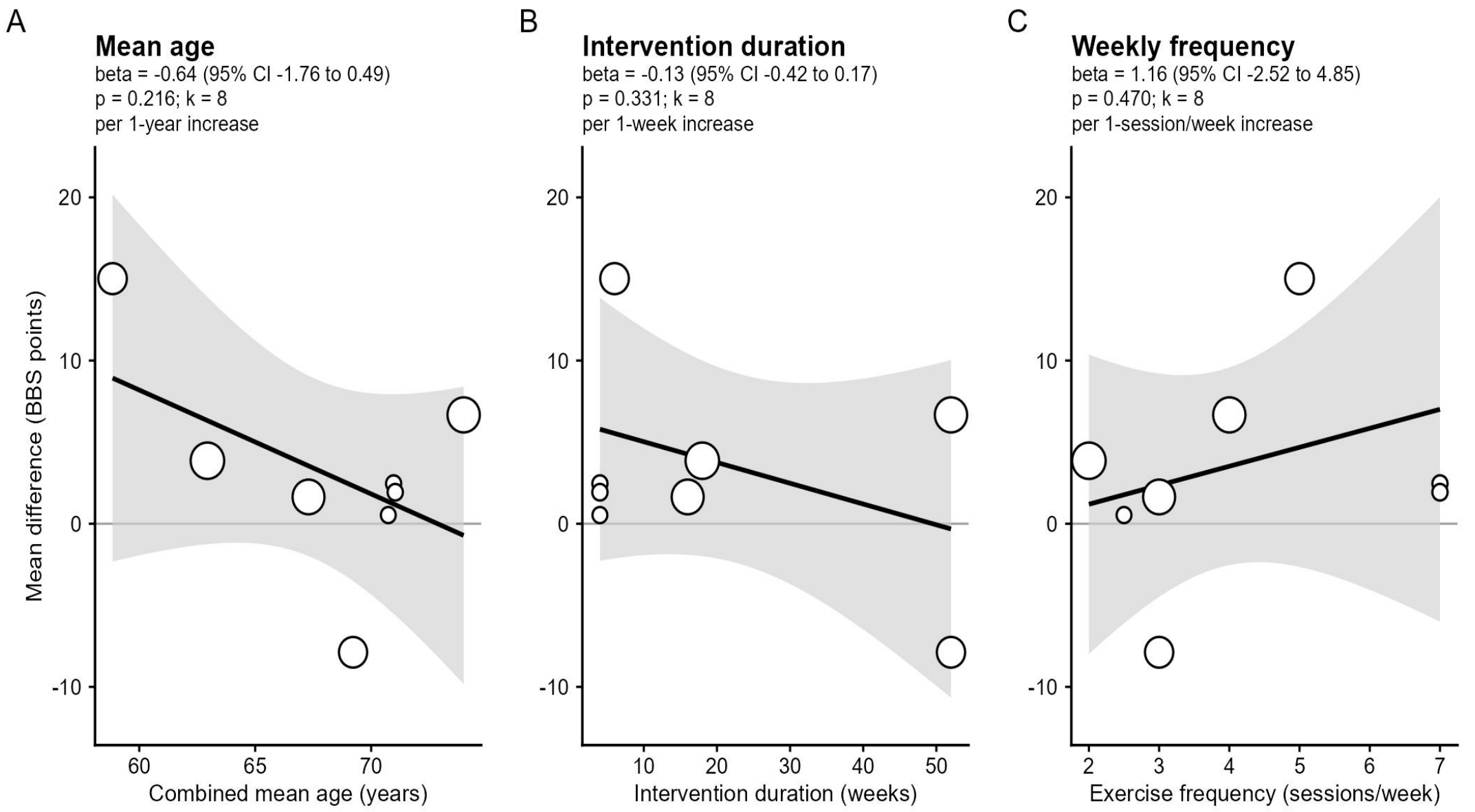

Figure S14. Univariable meta-regression analyses for BBS

Note. BBS, Berg Balance Scale; MD, mean difference; CI, confidence interval. Positive MD values favour exercise. Bubble size reflects inverse-variance study weight. Each moderator was analysed in a separate univariable random-effects meta-regression using REML and Hartung-Knapp inference. Shaded bands represent 95% confidence intervals for the fitted regression line.

6.3 30STS

Table S12. Univariable meta-regression analysis for 30STS

**Exploratory univariable meta-regression analyses for 30STS**							
**Moderator**	**Beta unit**	**k**	**Beta (95% CI)**	**P value**	**Pseudo-R2 (%)**	**Residual tau2**	**Residual I2 (%)**
Combined mean age	per 1-year increase	7	-0.31 (-0.53 , -0.09)	0.016	79.2	0.954	55.4
Intervention duration	per 1-week increase	8	0.09 (-0.19 ,0.37)	0.460	0	4.188	82.7
Weekly exercise frequency	per 1-session/week increase	7	1.66 (-3.12 , 6.43)	0.413	0	4.783	80.1

Session duration	per 10-minute increase	7	0.36 (-1.47 , 2.20)	0.631	0	5.234	84.6

Note. Each moderator was examined in a separate random-effects meta-regression using REML and Hartung-Knapp inference. Beta is the change in the post-intervention mean difference (exercise minus control) per unit shown. Positive beta values represent a shift toward greater exercise benefit for 30STS. Pseudo-R^2 is the proportion of between-study heterogeneity explained relative to the corresponding complete-case intercept-only model. All analyses are exploratory because of the small number of effect estimates. 30STS, 30-second sit-to-stand test; CI, confidence interval; REML, restricted maximum likelihood.

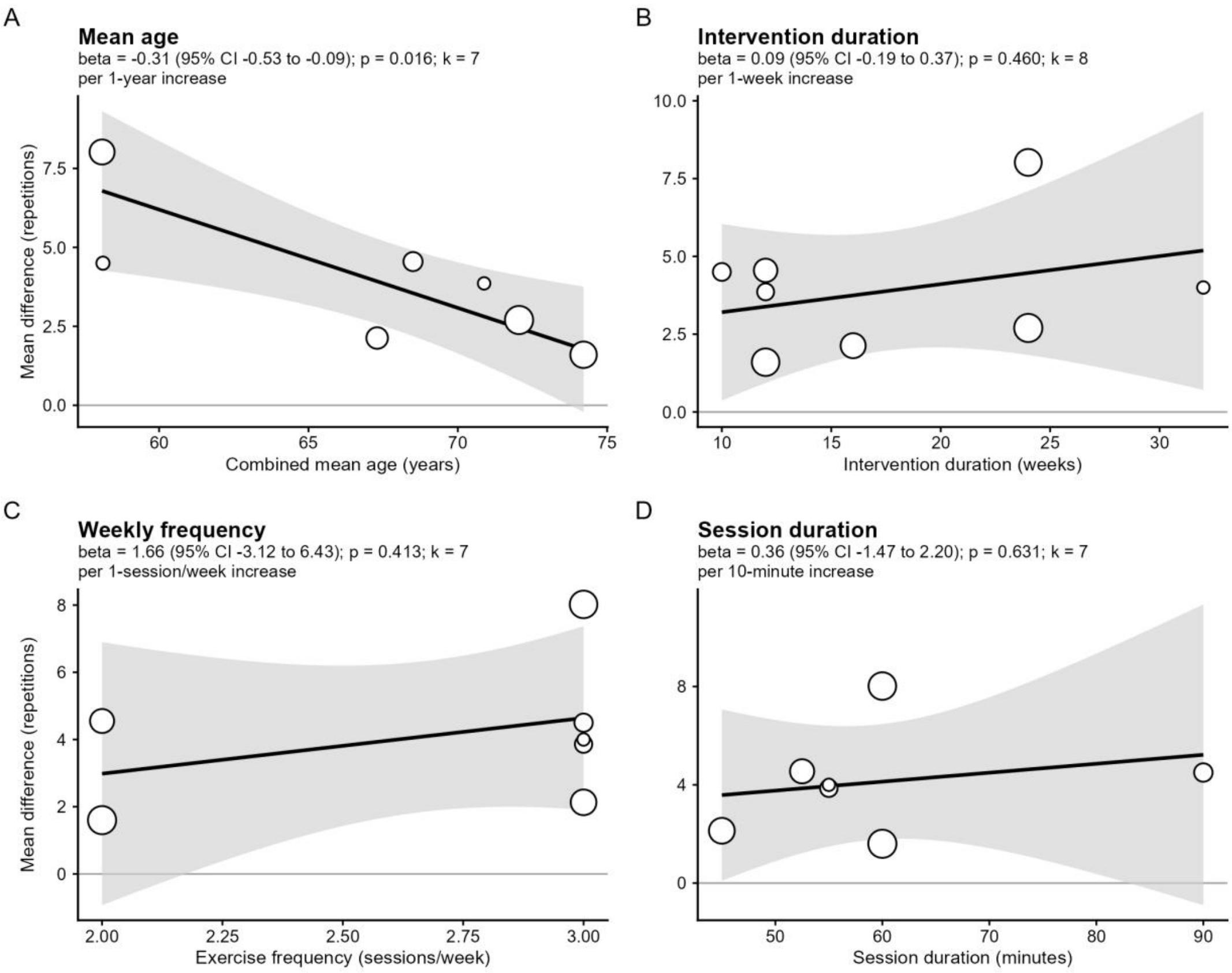

Figure S15. Univariable meta-regression analyses for 30STS

Note. 30STS, 30-second sit-to-stand test; MD, mean difference; CI, confidence interval. Positive MD values favour exercise. Bubble size reflects inverse-variance study weight. Each moderator was analysed in a separate univariable random-effects meta-regression using REML and Hartung-Knapp inference. Shaded bands represent 95% confidence intervals for the fitted regression line.
